# A Compendium of Nucleosome and Transcript Profiles Reveals Determinants of Chromatin Architecture and Transcription

**DOI:** 10.1371/journal.pgen.1003479

**Published:** 2013-05-02

**Authors:** Harm van Bakel, Kyle Tsui, Marinella Gebbia, Sanie Mnaimneh, Timothy R. Hughes, Corey Nislow

**Affiliations:** 1 Department of Genetics and Genomic Sciences, Icahn School of Medicine at Mount Sinai, New York, New York, United States of America; 2 Donnelly Centre for Cellular and Biomolecular Research, Toronto, Ontario, Canada; 3 Department of Pharmaceutical Sciences, University of Toronto, Toronto, Ontario, Canada; 4 Department of Medical Research, Banting and Best, Toronto, Ontario, Canada; 5 Department of Molecular and Medical Genetics, University of Toronto, Toronto, Ontario, Canada; 6 Department of Pharmaceutical Sciences, University of British Columbia, Vancouver, British Columbia, Canada; Medical Research Council Human Genetics Unit, United Kingdom

## Abstract

Nucleosomes in all eukaryotes examined to date adopt a characteristic architecture within genes and play fundamental roles in regulating transcription, yet the identity and precise roles of many of the trans-acting factors responsible for the establishment and maintenance of this organization remain to be identified. We profiled a compendium of 50 yeast strains carrying conditional alleles or complete deletions of genes involved in transcriptional regulation, histone biology, and chromatin remodeling, as well as compounds that target transcription and histone deacetylases, to assess their respective roles in nucleosome positioning and transcription. We find that nucleosome patterning in genes is affected by many factors, including the CAF-1 complex, Spt10, and Spt21, in addition to previously reported remodeler ATPases and histone chaperones. Disruption of these factors or reductions in histone levels led genic nucleosomes to assume positions more consistent with their intrinsic sequence preferences, with pronounced and specific shifts of the +1 nucleosome relative to the transcription start site. These shifts of +1 nucleosomes appear to have functional consequences, as several affected genes in Ino80 mutants exhibited altered expression responses. Our parallel expression profiling compendium revealed extensive transcription changes in intergenic and antisense regions, most of which occur in regions with altered nucleosome occupancy and positioning. We show that the nucleosome-excluding transcription factors Reb1, Abf1, Tbf1, and Rsc3 suppress cryptic transcripts at their target promoters, while a combined analysis of nucleosome and expression profiles identified 36 novel transcripts that are normally repressed by Tup1/Cyc8. Our data confirm and extend the roles of chromatin remodelers and chaperones as major determinants of genic nucleosome positioning, and these data provide a valuable resource for future studies.

## Author Summary

The genome in eukaryotic cells is packaged into nucleosomes, which play critical roles in regulating where and when different genes are expressed. For example, nucleosomes can physically block access of transcription factor to sites on DNA or direct regulatory proteins to DNA. Consistent with these roles, nucleosomes assume a stereotypical pattern around genes: they are depleted at the promoter region that marks the start of genes and assume a regularly spaced array within genes. To identify factors involved in this organization, we generated high-resolution nucleosome and transcriptome maps for 50 loss-of-function mutants with known or suspected roles in nucleosome biology in budding yeast. We show that nucleosome organization is determined by the combined effects of many factors that often exert opposing forces on nucleosomes. We further demonstrate that specific nucleosomes can be positioned independently within genes and that repositioning of nucleosomes at the start of genes may affect expression of these genes in response to environmental stimuli. Data mining of this extensive resource allowed us to show that general transcription factors act as insulators at diverging promoters to prevent the formation of cryptic transcripts, and also revealed 36 novel transcripts regulated by the Tup1/Cyc8 complex.

## Introduction

Chromatin is comprised of repeating units of nucleosome particles [1], [2] consisting of approximately 147 base pairs (bp) of DNA wrapped around a core histone octamer [3], [4]. The presence of nucleosomes and their relative occupancy on DNA can influence the access of proteins to DNA and they therefore play key roles in regulating DNA transactions such as replication and transcription [5]. Indeed, many of the effects exerted by transcriptional regulators in yeast and metazoans are mediated through interactions with regulators and coregulators that change the chromatin state to a more open (in case of activation) or closed conformation (in case of repression) [6]. Correspondingly, disruption of nucleosomes has a range of cellular consequences, including cryptic transcription [7], [8], which can result from the unmasking of sequences resembling promoters, or the alteration of histone marks that affect the function of accessory factors responsible for degradation of these transcripts [9]. Identifying both the cis and trans acting determinants of nucleosome occupancy and positioning is therefore key to more fully understand transcriptional regulation.

Genome-wide nucleosome profiling studies have revealed a high degree of organization around genes [10]–[13], with several conserved features: a nucleosome depleted region (NDR) immediately upstream of the transcription start site (TSS), followed by a regularly spaced array of nucleosomes across the gene body which then gradually dissipates towards the end of the gene [14] and, for many genes, ends with an NDR in the 3′ untranslated region. The average spacing of nucleosomes in *S. cerevisiae* is 165 bp, with a linker region of ∼18 bp separating adjacent nucleosomes [11], [14]. The location of the 5′ NDR coincides with the promoter region and is enriched for TF binding sites [11]. Its formation in yeast appears to be driven mainly by poly(dA:dT) tracts that are structurally rigid and refractory to nucleosome assembly [15]–[17], as well as by a small set of nucleosome-excluding transcription factors (TFs) such as Rsc3, Rap1, Abf1, and Reb1, which direct NDR formation at hundreds of promoters containing their binding motifs [18], [19]. Other factors such as the Tup1 and Cyc8 co-repressors have the opposite effect and induce the formation of closed chromatin at the promoters of the genes they repress [20]–[23]. Particular attention has been focused on the attributes of the +1 nucleosome which lies immediately downstream the 5′ NDR and which is thought to have a regulatory function by controlling transcription initiation [24], [25]. The +1 nucleosome has a well-defined position relative to the TSS [12], [14] and even small lateral movements of as few as 2–3 bp can (un)mask regulatory sites near the +1 nucleosome boundary [24].

In contrast to promoter NDRs, the determinants of genic nucleosomal organization are less well understood. *In vitro* nucleosome reconstitution studies with purified histones and DNA indicate that as much as half of all nucleosome positions may be determined by intrinsic nucleosome-DNA sequence preferences [26], [27], however, these experiments do not reproduce the typical nucleosome periodicity relative to the TSS observed *in vivo* [28]–[30]. One biophysical model to explain nucleosome positioning that does not rely on underlying sequence features is the statistical positioning model [31], which predicts that nucleosomes will form regularly spaced arrays relative to a genomic barrier (such as the NDR) due solely to steric hindrance between neighboring nucleosomes. Nucleosome organization *in vivo* shows several features consistent with statistical positioning [10], [14], [32], but *in vitro* profiles obtained using varying histone to DNA ratios do not [28], [33], indicating that other factors are required to explain genic nucleosome architecture *in vivo*. Indeed, a recent *in vitro* reconstitution study demonstrated that an ATP-dependent mechanism, presumably via the action of chromatin remodeling enzymes, is required to produce periodic nucleosome patterns. Based on these observations, an alternative model was proposed in which nucleosomes are actively stacked against the NDR barrier at the 5′ ends of genes [33]. These data strongly implicate protein factors in the organization of genic nucleosomes.

Several studies have highlighted the importance of remodeler ATPases in nucleosome organization around genes [13], [33]–[36]. Combined disruption of the remodeler ATPases Chd1 and Isw1 results in a total loss of patterning [37], suggesting that their coordinated action is critical for establishing *in vivo* chromatin architecture. Additional factors contribute to the regulation of genic nucleosomes, e.g. histone chaperones such as Spt16 and Spt6 are thought to affect genic nucleosome distribution by virtue of their role in histone turnover and nucleosome reassembly during transcription [7]. Loss of Spt6 results in decreased levels of genic nucleosomes and a loss of genic nucleosome organization [7], [38]. The FACT component Spt16 shares many of the phenotypic effects of Spt6 [7], [39], including widespread antisense transcription defects in genes [7], [8]. Changes in nucleosome spacing have also been observed in RNA polymerase II mutants [40], suggesting that transcription also promotes nucleosome organization [28], [40].

Here, we examined a compendium of 55 mutations and conditions in *S. cerevisiae* for their effects on nucleosome occupancy, positioning and transcription. Loss of the remodeler ATPases Chd1, Ino80 and Isw1, the CAF-1 complex (Rlf2, Cac2, Msi1) or Spt16 leads to significant displacement of 5,886 nucleosomes on 3,616 genes such that they assume positions that are more consistent with their intrinsic DNA-binding preferences. Most rearrangements of individual genic nucleosomes are within the linker regions, with little effect on neighboring nucleosomes, indicating that there is considerable positional flexibility within genic nucleosome arrays. Changes were most apparent at distal genic nucleosomes relative to the TSS; however, we also frequently observed repositioning of the +1 nucleosome, for example, upon histone depletion and in strains lacking Ino80 and Isw1. In the case of Ino80, selected genes with +1 nucleosome shifts involved in iron and glucose homeostasis showed altered gene expression responses in response to environmental stimuli. Changes in nucleosome occupancy and positioning were coupled to genome-wide transcription changes, giving rise to antisense transcripts in CAF-1 complex mutants, while loss of Reb1, Abf1, Tbf1, and Rsc3 function resulted in cryptic transcripts in the promoter regions of their target genes. Our findings demonstrate the utility of our compendium as a valuable resource for future studies.

## Results

### Compendium overview

**Figure 1:**
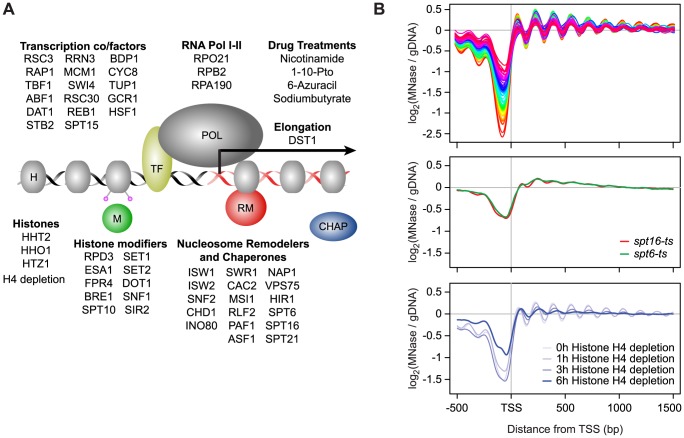
Global nucleosome occupancy profiles. A) Schematic overview of compendium mutants and conditions. B) Average nucleosome occupancy relative to the curated transcription start sites of 5,043 *S. cerevisiae* genes, expressed as the log2 ratio of probe intensities of MNase-treated nucleosomal DNA samples over un-crosslinked DNA samples. 52 mutants and conditions with a canonical nucleosome distribution are shown in the top panel and differentially colored according to average NDR occupancy. Mutants with disrupted global nucleosome occupancy profiles are shown below.

We examined 50 single-gene loss-of-function strains, comprised of gene deletions and temperature-sensitive (*ts*) or tetracycline promoter-shutoff (*tet*) alleles (Table S1). These genes were selected based on their known or potential role in nucleosome biology and included remodeler ATPases and chaperones, histones and histone modifiers, transcription and elongation factors, and components of RNA polymerase I and II (Figure 1A). The compendium also included 4 compounds targeting transcription and histone deacetylases, as well as a histone depletion time course performed with a strain in which H4 gene expression is exclusively under the control of a GAL1 promoter [41]–[44]. Glucose-induced repression leads to rapid nucleosome depletion in this strain. Genome-wide nucleosome occupancy profiles were generated using Affymetrix Tiling arrays with probes spaced every 4 bp [45], or next-generation sequencing. Identically prepared total RNA samples for each strain and treatment were analyzed on the same platform for strand-specific expression differences. Each compendium condition was compared to a matched wild-type (WT) reference grown in parallel (31 WT samples in total). To facilitate downstream analyses, we also prepared a manually curated set of transcript starts and ends for 5,043 yeast genes by using publicly available sequencing and tiling array data, as well as our compendium data. The nucleosome and expression profiles and gene annotations are available as a resource through the Nucleosome Compendium Browser at http://nbrowse.ccbr.utoronto.ca/mgb2/gbrowse/nucleosome/ and have been deposited in GEO.

Overall we find that most compendium mutants/conditions maintain a canonical nucleosome occupancy pattern with a depleted region (NDR) directly upstream the TSS and a regularly spaced nucleosome array across the gene when considered in aggregate (Figure 1B, Figure S1). The lack of dramatic response in most mutants is not necessarily surprising, based on a recent study which demonstrated that many chromatin mutants manifest their effects under conditions of stress [46], and may be explained in part due to inherent redundancy of factors involved in chromatin homeostasis. Each profile did, however, contain informative deviations from the WT reference. The greatest changes in nucleosome occupancy are seen for genes in the TF and nucleosome remodeler/chaperone categories. In the former category, the changes are predominantly localized to NDRs (Figure S2), which we previously showed is linked to the presence/absence of TF binding sites [18]. Changes in NDR occupancy in TF mutants are also generally correlated with expression changes of the genes with which these NDRs are associated (Figure S2). Nucleosome occupancy changes seen in mutants of nucleosome remodelers and histone chaperones occur more broadly throughout the genome, including within gene bodies and NDRs. Interestingly, the loss of histone modifiers resulted in only modest changes in nucleosome occupancy. Given that the compendium encompasses a broad range of modifiers, including those involved in histone (de)acetylation, methylation, phosphorylation, proline isomeration and ubiquitination, this observation suggests that any single histone mark plays a relatively minor role in regulating nucleosome occupancy, and by extension, genic nucleosome organization. We found 3 compendium conditions with severely disrupted nucleosome organization around genes (Figure 1B). Loss of the elongation factors Spt6 and Spt16 resulted in an almost complete loss of genic patterning, consistent with previous studies [7], [38]. We also found a progressive reduction of nucleosome patterning across the gene body within 3 to 6 hours after the shut-off of histone H4 transcription. The apparent increase in NDR occupancy after 6 hours is likely due to a normalization effect that makes NDR regions appear less pronounced as the result of a large global decrease in histone levels (see below). The fact that the global nucleosome organization around genes is maintained across most compendium conditions is consistent with cells employing redundant mechanisms that are robust against disruptions of individual chromatin modifiers and further underscores that maintenance of nucleosome occupancy is critical for cell fitness.

### Nucleosome linkers allow for positional flexibility of genic nucleosomes

In addition to the impact of gain or loss of nucleosomes, DNA-associated processes are also affected by repositioning of assembled nucleosomes, for example through modulation of regulatory site accessibility [47]. We therefore expanded our analysis to assess nucleosome shifts – defined here as a change in their average center position – relative to the TSS. To this end, we applied a modified Gaussian filter to our microarray and sequencing data to determine individual nucleosome positions [48], and calculated the degree of shift for each nucleosome in each compendium condition by subtracting its estimated center position from that of the nearest nucleosome in the corresponding WT reference, located within a 100 bp window (see Materials and Methods).

**Figure 2:**
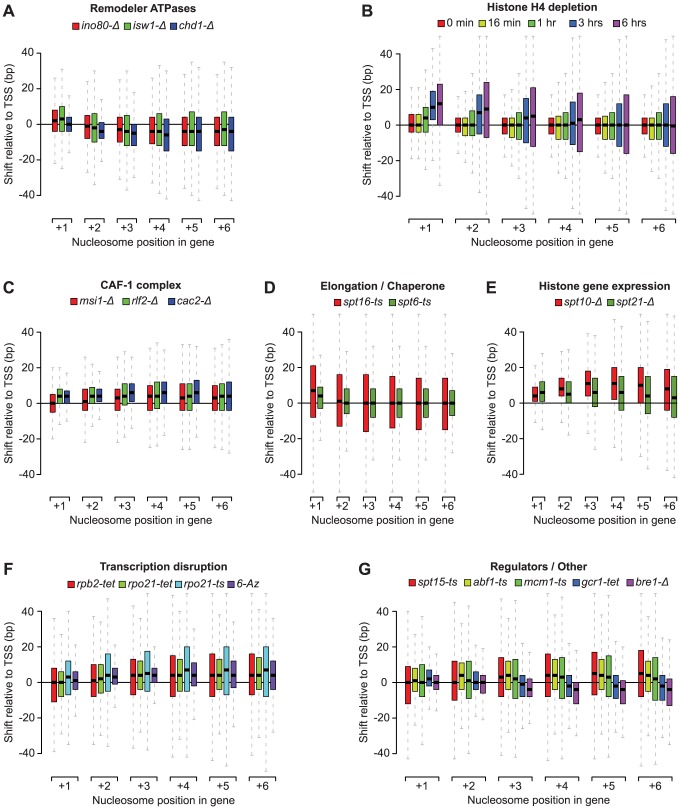
Profiles of genic nucleosome shifts. Box plots of nucleosome shifts for 20 conditions that show a median deviation of at least 4 base pairs for at least one genic nucleosome position, organized in 7 functional groups: A) remodeler ATPases, B) histone depletion, C) CAF-1 complex subunits, D) Elongation factors and chaperones, E) histone gene regulators, F) transcription disruption and G) all other factors. Boxes correspond to the spread between the upper and lower quartile, with medians indicated by a solid horizontal line, and whiskers extend up to 1.5 times the inter-quartile range. Nucleosome positions are relative to the transcription start site. Positive and negative numbers correspond to shifts away or towards the TSS, respectively.

Our nucleosome position analyses revealed striking patterns of genic nucleosome shifts relative to the TSS in 20 tested conditions, with median deviations ≥4 bp for at least one genic nucleosome position that were not seen in the WT reference profiles and other compendium conditions (see Figure S3 for comparison). We grouped these conditions into 7 categories based on shared biological function (remodeler ATPases, regulators of histone gene expression, the CAF-1 complex, elongation/chaperone) or experimental condition (histone depletion and transcription disruption) (Figure 2A–2F). Conditions that fell outside these broad classes were collapsed into a single category (Figure 2G) and included conditional loss-of-function mutants of the E3 ubiquitin ligase Bre1, as well as the essential transcription factors Spt15 (TBP), Abf1, Mcm1, and Gcr1. For the subset of essential genes, the observed effects may represent the combined effects of gene inactivation on chromatin and additional indirect effects. For conditions with published nucleosome occupancy maps, our nucleosome shift data is consistent with reported changes in genic nucleosome profiles. For example, a global reduction in transcription, either through loss of the RNAPII subunits Rpb2 or Rpo21, or by treatment with 6-Azauracil, which limits transcription elongation rates by reducing intracellular GTP levels [49], resulted in genic nucleosome movements away from the TSS (Figure 2F), consistent with the increased nucleosome spacing reported in Pol II mutants [40]. Likewise, we confirm genic nucleosome shifts in strains deleted for the remodeler ATPases Ino80, Isw1 and Chd1 (Figure 2A) [13], [34], [36].

The patterns of nucleosome shifts differed markedly between conditions, with the predominant effects being on either proximal or distal nucleosomes relative to the TSS, as well as a median shift towards or away from the TSS (e.g. compare Figure 2A and 2B). The progressive increase in the magnitude of shifts for nucleosomes further away from the TSS observed in many conditions is consistent with the additive effects of changes at more proximal positions. Most shifts ranged from 1 to 20 bp, with median deviations at each nucleosome position between 1 and 12 bp, placing them within the confines of inter-nucleosome linker regions, which average ∼18 bp in *S. cerevisiae* [11], [14, this study]. The range of lateral mobility suggests that although *in vivo* movements of genic nucleosomes in yeast are sterically limited by their neighbors, the linker regions do allow for positional flexibility. Notably, several conditions also exhibited hundreds of nucleosome shifts that exceeded the average size of the linker region, discussed below. We did not observe nucleosome shifts in a strain deleted for the non-essential the linker histone Hho1 (data not shown), indicating that this histone does not play a major role in setting inter-nucleosomal linker distance. This is consistent with the fact that Hho1 is present at much lower levels compared to core histones [50].

Taken together, our data suggest that remodeler ATPases, cellular histone levels, histone chaperones and transcription all exert distinct and sometimes opposite net effects on nucleosome positioning and inter-nucleosome spacing, and reveal considerable positional flexibility of genic nucleosomes. We next examined individual classes of rearrangements in more detail.

### Loss of chromatin remodeler ATPases repositions proximal genic nucleosomes with consequences for gene regulation

**Figure 3:**
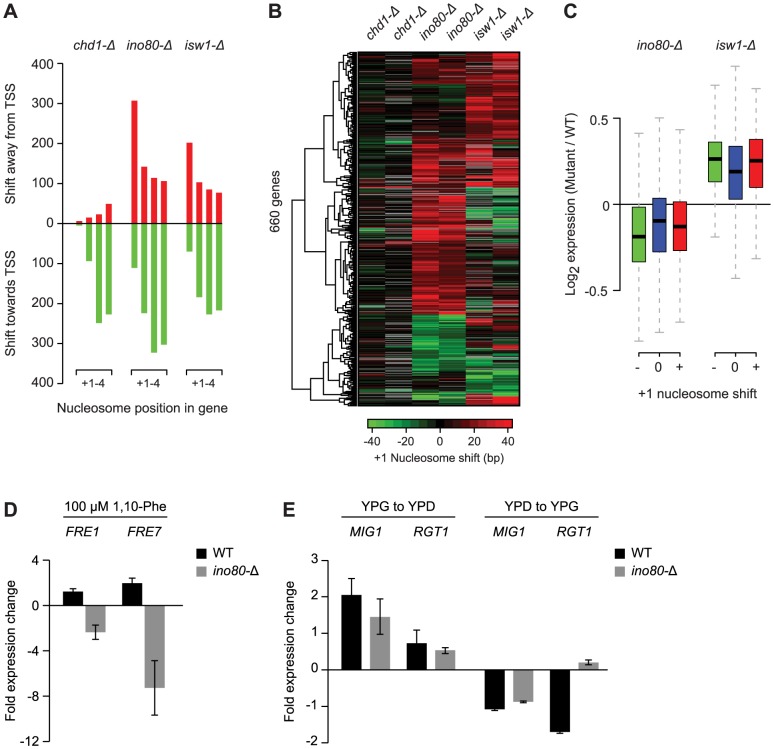
Loss of Ino80 or Isw1 leads to +1 nucleosome shifts at a subset of genes. A) Number of +1 to +4 nucleosomes with significant shifts in strains bearing deletions of the remodeler ATPases Chd1, Ino80 or Isw1, compared to 36 WT conditions. B) Hierarchical clustering of shifts at +1 nucleosomes found as significant in at least one of the remodeler ATPase mutants. Two biological replicates, each grown on different days, are shown for each mutant. The direction and degree of shift relative to the transcription start site is indicated by the color bar. Positions for which a shift could not be determined because of absent nucleosome calls in WT or mutant strains are colored grey. C) Box plot of the change in expression for genes with positive (red) or negative (green) +1 nucleosome shifts in *ino80-Δ* or *isw1-Δ* strains, compared to genes without shifts (blue). Box and whisker definitions follow those in Figure 2. D) qPCR analysis of *FRE1* and *FRE7* expression changes after 30 min treatment with 100 µM 1,10-phenantroline in a WT or *ino80-Δ* background. E) qPCR analysis of *MIG1* and *RGT1* expression changes upon shifts from YPG to YPD (left) or YPD to YPG (right) in a WT or *ino80-Δ* background.

Deletion of the remodeler ATPases Chd1, Isw1 and Ino80 generally moved genic nucleosomes closer to the TSS (Figure 2A), consistent with previous studies of these remodelers [34], [36], [37]. To better characterize the changes, we identified nucleosomes with a significant (T-test p<0.05) shift of at least 10 bp in two biological replicates compared to their position in the 31 wild-type strains that were independently grown and analyzed during the course of the study. In light of the emphasis in recent studies on the range of factors that contribute to the positioning of the +1 nucleosome, and the relative dearth of study on the +2, +3, and +4 nucleosomes [33], [36], we specifically examined changes at these positions. In each condition we identified hundreds of individual nucleosome rearrangements (Figure 3A), including many that exceeded the average inter-nucleosomal distance. At every position we find both positive and negative shifts relative to the TSS. Overall, we identified 3,147 genic nucleosomes with shifts between the +1 and +4 position in the three remodeler ATPases, affecting 2,379 genes. Strikingly, most of these changes are remodeler-specific, with only 2% of the rearranged nucleosomes found in more than one condition.

One of the most prominent observations from our analysis of individual nucleosome changes is the distinctive behavior of the +1 nucleosome in the *ino80-Δ* and *isw1-Δ* strains. While distal nucleosomes move predominantly *towards* the TSS, most +1 nucleosomes show a strong movement *away* from the TSS (Figure 2, Figure 3A), confirming other recent observations of these remodelers [36]. This displacement is particularly interesting as the +1 nucleosome has been considered the most well-positioned genic nucleosome, and whose position is primarily determined by the presence of fixed barrier elements such as poly(dA:dT) tracts [10]. We also observed +1 nucleosome shifts in several other conditions (Figure 2), however, the opposing effects on the proximal vs. distal nucleosomes were unique to *ino80-Δ* and *isw1-Δ* and prompted us to examine these mutants in greater detail. A hierarchical clustering of the degree of +1 nucleosome shifts (Figure 3B) shows that loss of Ino80 and Isw1 affects distinct sets of +1 nucleosomes and can sometimes result in opposite effects on the same nucleosome. Thus, there are key differences in both the magnitude and direction of +1 nucleosome shifts in Ino80 and Isw1 mutants; this is despite the observation that these factors have a high degree of co-occupancy at the 5′ ends of genes [36]. In contrast to Ino80 and Isw1, the Chd1 deletion mutant is characterized by an invariant +1 nucleosome position, with effects on distal nucleosomes limited almost exclusively to movements towards the TSS.

Given the proximity of the +1 nucleosome to the transcription start site and its potential role in regulating transcription, we assessed the effects of +1 nucleosome shifts in the Ino80 or Isw1 deletion mutants on gene expression. In standard growth conditions, genes with significant changes in +1 nucleosome position, either positive or negative, showed only minor changes in gene expression compared to genes with stable +1 nucleosomes (Figure 3C). We also did not find any correlation between the degree or direction of nucleosome repositioning, and transcription changes (data not shown). We attribute this to the fact that the effects of nucleosome repositioning may be activating or inhibitory, depending on local sequence context, e.g. on whether binding motifs of repressors or activators are (un)masked. To provide a more detailed assessment of the effects of +1 nucleosome position changes on individual genes, we asked if any of the affected genes had an altered environmental expression response. Among the 418 genes with shifted +1 nucleosomes in *ino80-Δ* strains, we identified 2 genes, *FRE1* (−25±15.0 bp shift) and *FRE7* (−15±4.2 bp shift), that are known to be induced in iron-limiting conditions [51], [52], as well as 2 glucose-responsive transcription factors, *MIG1* (+26±8.5 bp shift) and *RGT1* (+19±2.8 bp shift) [53], [54]. None of these genes showed significant shifts (>10 bp) at more distal genic nucleosome positions. Consistent with our hypothesis of a regulatory function for the +1 nucleosome, we find a complete loss of induction of *FRE1* and *FRE7* in an *ino80-Δ* background compared to WT strains, following treatment with the iron chelator and transcription inhibitor 1,10-Phenantroline (Figure 3D).Similarly, the response of MIG1 and RGT1 to changes in glucose levels is altered, with a marked loss of *RGT1* repression (vs. WT) upon a shift from YPD to YPG media (Figure 3E). These observations suggest that regulation of the position of the +1 nucleosome by Ino80 can affect gene expression at these loci and that these effects may have biological consequences, though this hypothesis remains to be further tested to rule out potential indirect effects of *INO80* deletion.

### Intrinsic DNA–sequence preferences drive nucleosome rearrangements in remodeler ATPase mutants

Several scenarios could account for the nucleosome rearrangements seen upon loss of remodeler ATPases. First, there could be specific associations between remodelers and individual genes or nucleosomes. To test this, we analyzed publicly available data [36], which indicated that the affected nucleosome positions are indeed bound by remodelers, but did not reveal any difference in Ino80 or Isw1 levels at positions with shifted nucleosomes compared to other nucleosome positions (Figure S4), suggesting that they are not preferentially targeted. Secondly, we considered that the rearrangements result from changes in the number of nucleosomes following the deletion or depletion of remodeling ATPases, by assessing changes in the levels of histone H3 as a proxy for changes in global nucleosome levels. Histone H3 levels were unchanged in *chd1-Δ*, but decreased by 12% and 17% in *ino80-Δ* and *isw1-Δ* strains, respectively (Figure S5). This suggests that the shifts of +1 nucleosomes away from the TSS in *ino80-Δ* and *isw1-Δ* strains, but not *chd1-Δ* strains, may be linked to changes in global nucleosome levels, though these changes cannot fully explain the shifts at more distal nucleosome positions we observed in all three strains.

**Figure 4:**
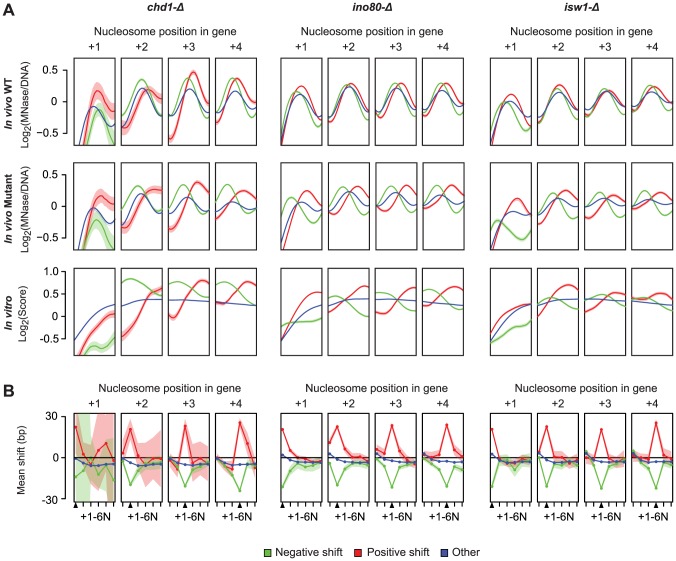
Nucleosomes disrupted in remodeler ATPase mutants are in more intrinsically preferred positions. A) WT (top row) and Chd1, Ino80 and Isw1 deletion mutant (middle row) occupancy profiles at genic nucleosome positions are shown in comparison to occupancy profiles (*in vitro* occupancy) based on intrinsic sequence preferences obtained from Kaplan et al. [27] (bottom row). Each panel corresponds to a genic nucleosome position, numbered relative to the TSS, and plots the *in vivo* or predicted occupancy for genes with a positive (red), negative (green), or no shift (blue) in nucleosomes at that position. Solid lines reflect the mean occupancy in a smoothing window with a bandwidth of 25 bp, with the corresponding 95% confidence interval indicated by shaded areas of the same color. B) Overview of the effects of nucleosome shifts at each +1 to +4 position on neighboring nucleosomes in the same genic array. Each panel shows the average shift profile (solid lines) and 95% confidence interval (shaded area) for all nucleosomes in the same array for genes with a positive (red), negative (green) or no shifts (blue) at the indicated genic nucleosome position (black triangle).

Finally, we considered the relationship between the nucleosome locations (relative to the TSS) affected by remodelers, and those locations on the DNA that are intrinsically preferred by nucleosomes, by comparing the *in vivo* profiles to *in vitro* nucleosome reconstitution data from Kaplan et al. [27]. Strikingly, upon loss of remodeler ATPase activity, there is widespread repositioning of nucleosomes towards more preferred DNA sequences (Figure 4A), which suggests that Chd1, Ino80 and Isw1 may act to disrupt these interactions to favor their organization into genic arrays. Given the potential for steric effects on neighboring nucleosomes we also examined how nucleosome shifts at each of the +1–4 positions affected other nucleosomes in the same genic array. Interestingly, while significant shifts at each genic nucleosome position were coupled to shifts of directly neighboring nucleosomes, these changes did not propagate to more distal positions in the same nucleosome array (Figure 4B). Indeed, most genes with a significant positive shift at proximal positions still show a negative shift at more distal genic nucleosomes. This further demonstrates the positional flexibility of genic nucleosomes and suggests that remodeler ATPases can exert opposite directional forces on proximal and distal genic nucleosomes.

### Depletion of histones results in distinct profiles of nucleosome repositioning

Using a promoter shutoff strategy [41]–[44], we found that histone H4 depletion (3–6 hours) led to a progressive shift of the +1, +2, +3 and +4 nucleosomes away from the TSS, with the most pronounced effect at the +1 position (Figure 2B). More distally, there was no net change, however, the greatly increased positional variance, (Figure 2B), suggested large shifts of individual nucleosome in both directions. Indeed, when using the criteria described above, we find equal numbers of significant nucleosome shifts ≥10 bp in both directions at the +3 and +4 positions (Figure S6). Within 3 to 6 hours after shutoff of H4 transcription, there is a 15% and 76% reduction in global histone levels (Figure S5), respectively, and a decrease of 18% and 27% in assigned nucleosome positions in our tiling array data at the Gaussian score thresholds used. As we observed for the remodeler ATPases, nucleosome loss led to rearrangement of nucleosomes to positions that are more consistent with their intrinsic DNA-sequence preferences (Figure S6), suggesting that steric hindrance by neighboring nucleosomes counteracts nucleosome sequence preferences, which is consistent with findings based on histone H3 depletion [55].

We also see a reduction in global histone levels upon deletion of the histone gene regulators Spt10 (19%) and Spt21 (19%) (Figure S5), in line with the reduction of histone gene expression reported in these conditions [56]. The *spt10-Δ* strain in our compendium showed reduced (>3.5-fold) expression at the HTA2-HTB2 locus, encoding histones H2A/H2B, as well for as the redundant HHF1-HHT1 and HHF2-HHT2 loci (>2-fold) encoding histones H3/H4. In the *spt21-Δ* strain, the HTA2-HTB2 (>2-fold) and HHF2-HHT2 loci (>1.4-fold) were affected. Surprisingly, loss of the histone gene regulators Spt10 and Spt21 led to some of largest rearrangements of genic nucleosomes in the compendium (Figure 2E) despite only a modest loss of nucleosomes (Figure S5). In the case of *spt10-Δ*, these changes are consistent with previous reports of global disruption of chromatin structure in this mutant [57]. There are other marked differences in the nucleosome shift profiles: while prolonged depletion of histone H4 alone results in +1 nucleosome shifts, loss of Spt10 and Spt21 predominantly affects distal nucleosomes. Given that Spt10 and Spt21 regulate the levels of histones H2A, H2B and H3, in addition to histone H4, this may indicate that cells respond differently to the concerted depletion of all four histone types. Alternatively, both Spt10 and Spt21 have recently been described to affect silencing at telomeres in a manner independent of changes in histone levels [58]; our findings are therefore also compatible with potential roles for Spt10 and Spt21 in regulating global chromatin structure that go beyond the regulation of histone gene expression.

Our observations of nucleosome shifts upon histone depletion differ from those of a previous study that reported no redistribution of nucleosomes along DNA in yeast *nhp6* mutants bearing deletions of *NHP6A* and *NHP6B*, despite a 20–30% reduction in histone levels [59]. Furthermore, a recent *in vitro* nucleosome reconstitution study in which whole cell extracts with additional ATP were used to reproduce *in vivo* patterning also did not find global changes in the spacing of +1–4 nucleosomes upon reduction of the histone∶DNA ratio by 50% [33]. The discrepancies between our findings and these studies may partially be accounted for by differences in the degree of nucleosome depletion between studies and in experimental setup (i.e. *in vitro* reconstitution vs. *in vivo* depletion). In addition, these studies focused primarily on large-scale rearrangements and may have missed smaller scale effects. Indeed, our reanalysis of the data for both studies shows similar shifts of the +1–4 nucleosomes upon reduction of histone levels (Figure S7), in particular for the reconstitution study [33], suggesting that with increased resolution the apparent discrepancy disappears, consistent with a recent histone H3 depletion study [55]. Taken together, these data show that there is greater positional flexibility of genic nucleosomes in response to changes in histone levels than previously assumed, with potential consequences for gene regulation.

### The CAF-1 complex contributes to genic nucleosome positioning and suppression of antisense transcripts

There is an increasing shift of distal genic nucleosomes away from the TSS upon loss of the CAF-1 subunits Msi1, Rlf2 and Cac2 (Figure 2C). The CAF-1 complex is involved in nucleosome assembly [60], [61] and has also been suggested to play a role in transcription [62], [63]. The nucleosome shifts in the CAF-1 mutants may be partially due to a loss of genic nucleosomes, as there is a decrease in global histone levels in the *msi1*-Δ (9%) and *cac2*-Δ (19%) strains (Figure S5). Nevertheless, the CAF-1 profiles are distinct from those obtained after prolonged H4 depletion, with an increased shift of distal nucleosomes in the former, compared to proximal nucleosomes in the latter. In addition, the differences in the CAF-1 profiles compared to Spt6 and Spt16 (Figure 2D), suggest that the CAF-1 complex plays a distinct role in organizing genic nucleosomes. As in other compendium conditions, nucleosome position changes in CAF-1 complex mutants appear to be partially driven by their intrinsic DNA-binding specificity (Figure S8), thus the CAF-1 complex may be added to the roster of factors that oppose intrinsic nucleosome positioning *in vivo*.

**Figure 5:**
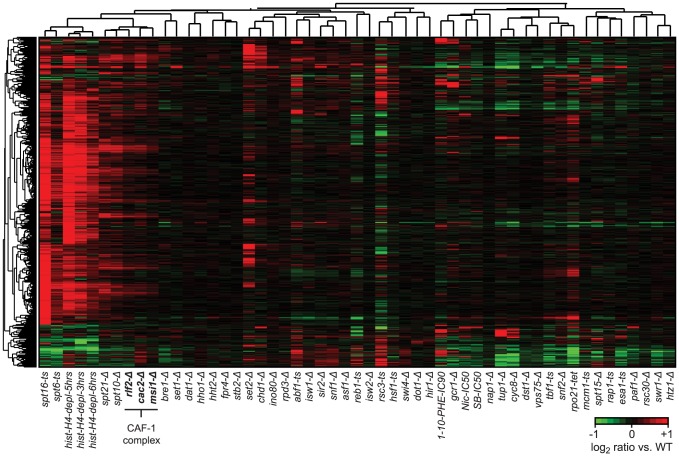
Cryptic antisense transcripts in CAF-1 complex mutants. Hierarchical clustering of average expression changes in 3,578 antisense (AS) regions. The set of AS transcripts was compiled by first identifying all AS transcripts with a more than 2-fold change in expression and a p-value<0.05 over at least 80 bp (10 probes) in at least one compendium condition, and then combining these transcripts in a non-redundant union set. Four strains (*rpa190-tet*, *rpb2-tet*, *rrn3-tet*, *bdp1-tet*) were excluded from the analysis as the large decrease in gene expression and/or global changes in total RNA composition in these strains made them unsuitable to our tiling array normalization procedure. Profiles corresponding to deletions of subunits of the CAF-1 complex are indicated in bold.

Previous studies of Spt6 and Spt16 mutants revealed that disruption of genic nucleosome arrays results in the expression of cryptic antisense transcripts (i.e. transcripts complementary to mRNAs) [7], [8]. We therefore sought to determine whether the loss of CAF-1 complex components results in similar transcription defects by examining all compendium conditions for a significant change in expression levels (≥2-fold; p≤0.05) for RNAs of at least 80 nt in length (Table S2) on the antisense strand of annotated genes. Hierarchical clustering of expression changes in all regions associated with antisense transcripts show, as expected, a large increase in antisense transcripts in the Spt6 and Spt16 mutants and upon histone depletion (Figure 5). There is also an increase in antisense transcripts in the *spt10-Δ* and *spt21-Δ* mutants, consistent with the down-regulation of histone gene expression and changes in nucleosome spacing we observe under these conditions. Loss of the CAF-1 complex components Rlf2, Cac2 or Msi1 leads to widespread cryptic antisense transcripts, albeit to a lesser extent. The individual deletions of these components each resulted in similar antisense transcription profiles that cluster with the *spt10-Δ* and *spt21-Δ* strains. These data confirm that the CAF-1 complex plays a role in genic nucleosome positioning and functions to prevent cryptic antisense transcription, in a manner distinct from Spt6 and Spt16.

### Loss of Abf1, Reb1, Rsc3, or Tbf1 results in cryptic transcripts at target promoters

**Figure 6:**
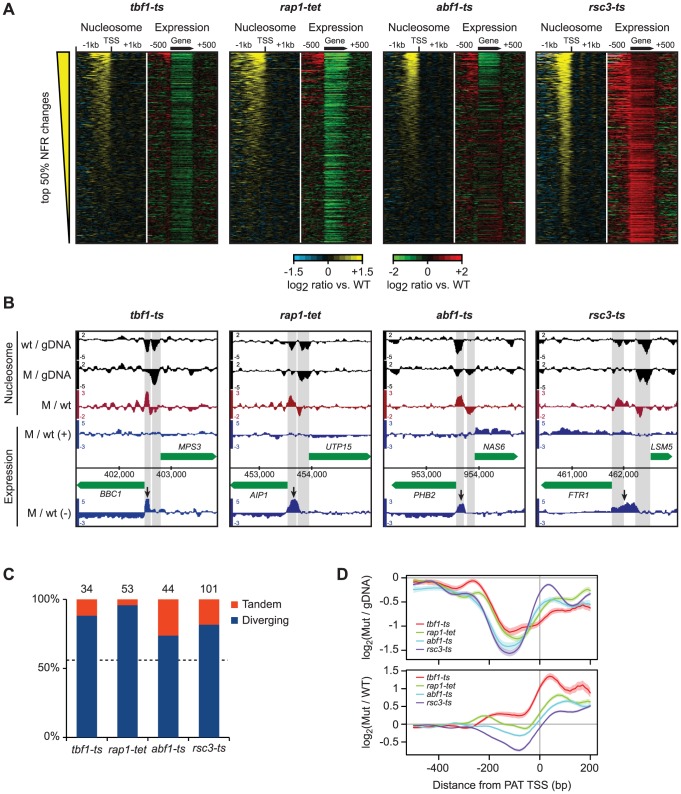
Disruption of nucleosome-excluding general transcription factors results in cryptic promoter transcripts. A) Correlation between changes in promoter nucleosome occupancy (left panels, yellow/blue) and transcription changes across the gene body and 1 kb intergenic flanking regions (right panels, red/green). In each panel, genes are ranked according to the average change in NDR nucleosome occupancy. NDRs were defined as the 200 bp region directly upstream of curated transcription start sites. B) Representative examples of promoter transcripts in each TF mutant (arrows). Positions of NDRs are highlighted in grey. C) Fraction of diverging and tandem genes among promoters that show significant changes in nucleosome occupancy and expression of cryptic transcripts. The number of promoter regions considered is indicated above each bar. D) Top: nucleosome occupancy relative to transcript starts for promoter transcripts in the *tbf1-ts*, *rap1-tet*, *abf1-ts* and *rsc3-ts* mutants. Bottom: changes in nucleosome occupancy compared to wild-type strains.

We identified 9,471 distinct regions with significant transcription changes in at least one compendium condition that did not overlap annotated strands of known features, totaling 7.0 Mb (29%) of the two strands of the genome sequence (24 Mb). The bulk of these regions were antisense to known genes (Figure 5, Table S2); however, we also observed many additional transcription changes in intergenic regions (Table S2). The breadth of these changes greatly expands on previous studies and provides a rich repository for subsequent studies. Here, we focused on transcripts originating in genomic regions that manifested changes in nucleosome positions and/or levels and found a strong link between changes in NDR nucleosome occupancy and the appearance of transcripts at the 5′ ends of genes in Tbf1, Abf1, Rsc3 and Rap1 mutants (Figure 6A), consistent with their roles in nucleosome exclusion at promoters [18], [64], [65] (Figure 6B). Other conditions in the compendium that showed strong changes in either NDR nucleosome occupancy and/or gene expression, e.g. histone depletion (Figure S9), did not show this effect, indicating that the appearance of these transcripts is a direct effect of the loss of the TFs. Given their location, we designated these transcripts as promoter associated transcripts (PATs). A complete list of all PATs and the genes they are associated with is provided in Table S3.

PATs share characteristics with cryptic unstable transcripts (CUTs) detected after disruption of the exosome complex [66], [67], such as their occurrence at gene termini. Moreover, many CUTs result from bidirectional transcription [66], [67] and similarly, we see that PATs are enriched at divergently transcribed genes (Figure 6B, Figure 6C), and originate from the NDRs of the upstream genes (Figure 6B, 6D). This suggests that most PATs in the TF mutants either result from an increase in divergent transcription from upstream promoters, or a failure to degrade these transcripts. Taken together, we conclude that the proper architecture of nucleosomes on the affected regions, established by the actions of Tbf1, Abf1, Rsc3 or Rap1, is important for preventing expression of cryptic promoter associated transcripts.

### A nucleosome signature reveals novel transcripts repressed by Tup1/Cyc8

**Figure 7:**
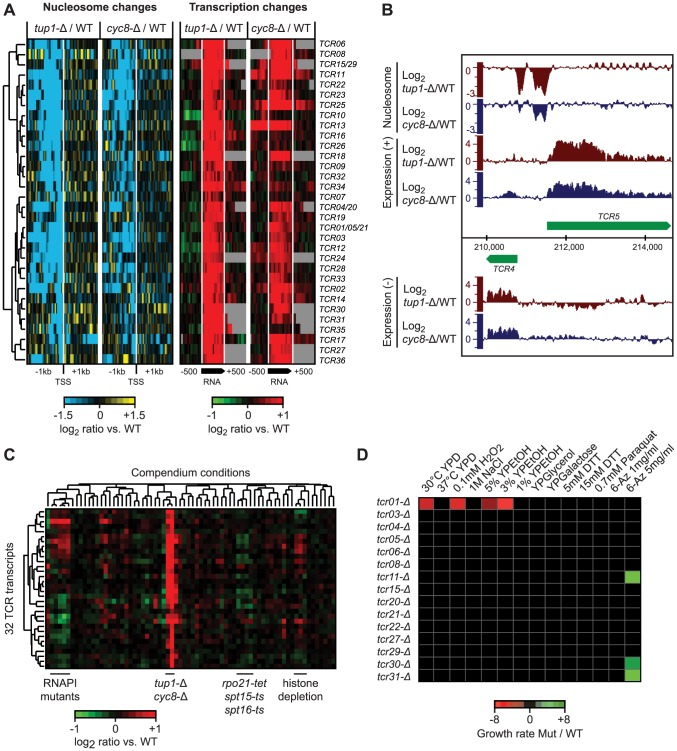
Novel transcripts identified in *tup1-Δ* and *cyc8-Δ* strains. A) Hierarchical clustering of 36 novel Tup1/Cyc8-repressed transcripts (TCR) showing changes in nucleosome occupancy directly upstream the transcription start site (blue/yellow; left) and expression changes across each transcript and 500 bp intergenic flanking region (red/green; right). The degree of change is indicated by the color bars. B) Example of de-repressed novel transcripts (*TCR4* and *TCR5*) in the *tup1-Δ* strain. C) Hierarchical clustering of average expression changes at the 36 TCR loci across all 55 compendium conditions. D) Results of phenotype analysis of 15 *tcr* deletion mutants in a *tup1-*Δ background across a panel of 14 different stress conditions. Growth rates were measured by spot assay and quantified using ImageQuant. Differences in growth rate between *tup-Δ* and *tup-Δ/tcr-Δ* strains are indicated by the color bar.

We identified the Tup1 and Cyc8 single deletion mutants as having a particularly strong relationship between changes in 5′ NDR occupancy and the expression of their target genes; almost all genes that showed an increased NDR depletion had increased expression, and vice versa (Figure S1). This prompted us to scan the genome for new transcripts with the same characteristics, to identify novel Tup1/Cyc8 regulated genes. Requiring a significant change in expression (≥2 fold; p≤0.05) across at least 80 nt, coupled with a 1.5-fold decrease in nucleosome occupancy 200 bp upstream, we identified 36 regions with significant changes in expression levels and the formation of a 5′ NDR when Tup1 and/or Cyc8 were deleted (Figure 7A, Table S4). We have designated these Tup1/Cyc8 repressed (TCR) transcripts. An example of two divergent loci identified in the subtelomeric region of chromosome 1 is shown in Figure 7B. Further examination of the 36 TCR loci in all compendium conditions confirmed that these transcripts are specifically regulated by Tup1 and Cyc8 (Figure 7C).

Among the 36 new transcripts we identified, 14 originate in subtelomeric regions, 13 are antisense to protein-coding genes and the remaining 9 are intergenic (Table S4). Seven of these transcripts overlap previously identified disabled ORFs (*TCR1*, *TCR21*, *TCR5*, *TCR4*, *TCR20*, *TCR15* and *TCR29*) [68]. The subtelomeric *TCR1*, *TCR4-6* and *TCR20-21* bear striking similarity to flocculation genes, however, the presence of multiple stop codons and indels suggests that they no longer encode functional proteins. Translated blast analysis (blastx) identified *TCR27* as potentially encoding a salt tolerance protein (COS3 hit, E-value 3e-30) and distant hits for *TCR7*, *TCR8*, *TCR22*, and *TCR30*, indicating that these five TCRs may be protein-coding (pseudo)genes. Finally, several of the intergenic transcripts we identified are divergently oriented relative to promoters of neighboring protein coding genes targeted by Tup1/Cyc8 (Table S4) and could therefore be the result of bidirectional transcription.

To determine if the novel Tup1/Cyc8 regulated transcripts are functional, we deleted 10 subtelomeric TCR genes and four others found in intergenic regions in a *ura8-*Δ (the “WT” reference) or *tup1-*Δ background, subjected them to a panel of 14 different stress conditions, and assessed changes in growth by serial dilution assays. The results of these assays are summarized in Figure 7D. As expected from their repression in WT conditions, none of the deletions in the *ura8-*Δ background showed a growth phenotype in any of the stress conditions. In contrast, several deletions exhibited growth defects in the *tup1-*Δ background (*tup1-Δ/tcr1-Δ* was 2.6, 2.8 and 6.1 times more sensitive in YPD, 0.1 mM H_2_O_2_ and 3% YPEtOH, respectively) or increased resistance (*tup1-Δ/tcr11-Δ*, *tup1-Δ/tcr31-Δ* and *tup1-Δ/tcr30-Δ* were 3.2, 3.3 and 2 times more resistant in 5 mg/ml 6-Azauracil, respectively) (Figure 7D), indicating that several of the loci we identified are functional.

## Discussion

In this study we find, by virtue of mutational inactivation and biochemical perturbation, multiple factors that act in concert to disrupt intrinsic nucleosome binding preferences and maintain *in vivo* positioning, and often exert opposing net forces on positioning. We report new roles in genic nucleosome positioning for Spt10, Spt21 and the CAF-1 complex, increasing the number of chromatin modifiers involved in nucleosome organization around genes. While the factors examined in this study predominantly affect different subsets of nucleosomes and genes, their loss generally leads to displacement of nucleosomes towards more intrinsically preferred sequences. Together these observations underscore the multiple ways in which cells actively counteract intrinsic nucleosome binding preferences in genic regions to achieve chromatin “homeostasis”. This holds true even for the +1 nucleosome, whose placement at the 5′ end of genes is considered extremely stable. Many of these perturbations of nucleosomes led to widespread transcription defects, which we captured by performing parallel genome-wide transcript analysis.

Of all the models that aim to capture the principles of nucleosome organization, the barrier-packing model [33] is the most comprehensive in that it accounts for most of the data published thus far. This model posits that nucleosomes are actively stacked against the TSS and was motivated by the observation that *in vitro* reconstitution of chromatin with reduced histone levels mainly affect the position of distal genic nucleosomes, with little effect on spacing and positioning of proximal nucleosomes [33], [59]. Our data, in contrast, reveal significant reorganization of proximal nucleosomes after depletion of histone H4 *in vivo*, including a pronounced shift of +1 nucleosome away from the TSS. Although this observation is not necessarily incompatible with a packing model (e.g. it could reflect a reduction in packing efficiency due to nucleosome loss), it does indicate that there are additional forces acting on proximal nucleosomes which oppose TSS stacking. Such opposing forces are also apparent in the variability in nucleosome shifts among many of the compendium mutants, with some increasing and others decreasing packing against the TSS. The overall movement of distal nucleosomes in Chd1, Ino80 or Isw1 loss-of-function mutants makes it unlikely that these remodelers are responsible for packing distal nucleosomes against the TSS, although roles for other remodeler ATPases such as Isw2 [36] are not excluded. We do find an expansion of nucleosome arrays upon loss of the CAF-1 complex, as well as Spt10 and Spt21, indicating that chaperones may contribute to packing against the TSS. Accordingly, TSS packing of nucleosomes appears to be the net outcome of multiple opposing forces, rather than the actions of any single class of factors.

The TSS packing model further predicts that shifts of proximal nucleosome should propagate throughout the array of genic nucleosomes. Although we find that local shifts can affect the positions of adjacent neighboring nucleosomes, these effects rarely spread to more distal nucleosomes. Indeed, many of the genes with a strong displacement of the +1 nucleosome away from the TSS in Ino80 and Isw1 still show an overall movement towards the TSS for more distal nucleosomes. A likely explanation for this effect is that nucleosome shifts can be buffered by changes in linker region length, which allows for a degree of local flexibility in genic arrays and decoupling of proximal and distal genic nucleosome positioning.

Within arrays, individual nucleosome shifts appear to be mainly driven by underlying sequence, with nucleosomes moving to positions that are more consistent with their intrinsic DNA-sequence preferences upon loss of Ino80, Isw1, Chd1, the CAF-1 complex, histone H4, Spt6 and Spt16 (data not shown). This extends observations previously made for Isw2 and reductions in histone H3 levels [55], [69], and suggests that there are many factors involved in disrupting intrinsic DNA sequence preferences. The movements of +1 nucleosomes away from the TSS we observed in many conditions are likely driven by the strong nucleosome-excluding properties of the NDR and confirm that many of these nucleosomes are not in their optimal intrinsic positions [28], [33], [69]. Taken together, our observations indicate that nucleosome packing is not unidirectional and strongly suggests that genic nucleosomes can and do position themselves independently.

In the perturbations interrogated here, the loss and/or repositioning of nucleosomes appears to drive the appearance of the cryptic transcripts in the parallel transcriptome maps. For example, the increased genic nucleosome spacing in strains bearing deletions of Spt10, Spt21 and components of the CAF-1 complex is accompanied by an increase in antisense transcripts. The patterns of antisense transcripts are similar to those observed in spt6 and spt16 mutants and upon histone H4 depletion, albeit to a lesser degree. The CAF-1 effects on genes are likely direct – the Msi1, Rlf2 and Cac2 subunits have been shown to be recruited to *PMA1* in a transcription-dependent manner, with patterns of association with the gene body resembling those of Spt6 and Spt16 [62]. Loss of the nucleosome-excluding transcription factors Tbf1, Rap1, Abf1 and Rsc3 results in cryptic transcripts at their target promoters, most of which appear to be the result of diverging transcription from upstream promoters. These findings are consistent with the transcriptional interference previously reported upon loss of an Abf1 binding site at the ARO4/HIS7 locus [70]. Interestingly, all four of these general regulatory/transcription factors have been shown to act as strong insulators that enable neighboring chromatin domains to be regulated independently [65], [71], and further, promoters containing combinations of these binding sites are proposed to act as insulators [65]. Our results suggest that the actions of nucleosome-excluding TFs can indeed establish a boundary between adjacent promoters that prevents diverging transcription.

The discovery of novel transcripts regulated by Tup1/Cyc8 shows that new genes and/or pseudogenes can still be found, even in the well-studied budding yeast genome and transcriptome. Several of these transcripts in subtelomeric regions bear strong similarity to flocculation genes, but the presence of multiple stop codons suggests that they no longer encode for functional proteins. A blast analysis of related yeast strains shows that, in contrast to the *S. cerevisiae* S288c reference strain (an acknowledged evolutionary outlier [72]), many *S. paradoxus* and *S. cerevisiae* isolates bear functional copies of these genes (data not shown). Combined signatures of chromatin modifications and changes in transcript levels have been successful in identifying new functional RNAs and our results indicate that the same principles can be used to identify new functional transcripts in yeast.

Our study has focused on a core set of chromatin modifiers, transcription factors and histones, yet even within this small set we identified several new factors that impact genic nucleosome architecture and transcription. By extrapolation, it is therefore likely that many other factors that play a role in nucleosome positioning remain to be identified. Crucially, there is still a lack of understanding of the interplay between nucleosome modifiers and chaperones and how they coordinately regulate and control nucleosome architecture at genes. For example, reported differences in nucleosome positioning and spacing between species, with their attendant effects on evolution [73], may well be a reflection of differences in the balance between these factors. The data provided here should prove very useful to begin classifying the contributions of these various factors.

## Materials and Methods

### Microarray design

Microarrays were designed in collaboration with Lars Steinmetz and Ron Davis at the Stanford Genome Technology Center [45] and Affymetrix (PN 520055) and contain one set of 25-mer probes spaced every 8 bp covering the Watson strand and a second set of probes, offset 4 bp from the first set, covering the Crick strand of the *Saccharomyces cerevisiae* S288c genome. The design allows for 8- or 4-bp resolution hybridizations of single- or double-stranded samples, respectively.

### *S. cerevisiae* culture conditions

The strains used in this study are listed in Table S1. For deletion strains, 5 mL cultures were grown overnight at 30°C in YPD. Cells were diluted to OD600 0.2/mL in 400 mL of YPD media the next morning, and grown to mid-log phase (OD600 0.8–1.0/ml) in 1 L flasks at 30°C while shaking, at which point RNA and nucleosomal DNA were isolated. All OD measurements were made using an Eppendorf BioPhotometer (SN#6131). Temperature-sensitive strains were grown at 22°C (permissive temperature) until mid-log phase. An equal volume of hot medium was then added to rapidly equilibrate the culture to 37°C (restrictive temperature), followed by a further 3 to 7 hours incubation at this temperature until a difference in OD between the mutant strain and its corresponding wild-type control became apparent. For Tet promoter-shutoff strains [74], doxycyline was added at 10 µg/ml for 24 h in order to obtain down-regulation for the gene of interest. Cells were then diluted to an OD600 of 0.2 and grown in the presence of doxycycline (10 µg/ml) until mid-log phase. These conditions were previously shown to lead to effective ablation of proteins encoded by essential genes [74].

For the histone depletion time course, the UKY403 strain harboring a deletion of both Histone H4 genes (HHF1, HHF2), a plasmid with the GAL1 promoter driving expression of Histone H4 (HHF2) and a plasmid with the GAL1 promoter driving expression of Histone H4 (HHF2), was grown in YP with 2% galactose until mid log phase [41]–[44]. Cells were then collected by centrifugation and transferred to YPD for 0, ¼,1, 3, 5, and 6 hrs. After each time point, samples were taken for nucleosome and expression analysis.

The haploid yeast strain BY4741 (parental strain of the haploid yeast deletion collection) was used for drug treatments. Cells were grown overnight and then diluted to an OD600 of 0.4. The drugs Nicotinamide (82 mM, IC50), Sodium Butyrate (20 mM, IC50), 1,10-Phenanthroline (100 µM, IC90) or 6-Azauracil (6 mM, IC90), all obtained from Sigma-Aldrich, were added to the media for 2 hours. Drug concentrations were predetermined to inhibit growth by 50% and/or 90%.

### Nucleosomal DNA isolation

Nucleosomal DNA was prepared according to Lee et al. [11] with a modified fragment size selection step. Briefly, cells were crosslinked by direct addition of methanol-free formaldehyde (Polysciences) to a final concentration of 2% for 30 min while shaking at 30°C. The reaction was quenched by adding glycine to a final concentration of 125 mM for 5 min. Cells were pelleted, washed with 20 mL phosphate buffered saline solution once, and resuspended in 6 mL of [1 M sorbitol, 50 mM Tris 7.4] with freshly added 10 mM β-mercaptoethanol in a 15 mL conical tube. Zymolyase (20T, TakaRa Biotechnology Co., Ltd, Japan) was added to a final concentration of 0.25 mg/mL and cells were spheroplasted at 30°C while gently rolling for 30 min. After zymolyase treatment, cells were pelleted and resuspended in 4 mL of [1 M sorbitol, 50 mM NaCl, 10 mM Tris 7.4, 5 mM MgCl_2_, 1 mM CaCl_2_, 0.075% NP-40] with freshly added 1 mM β-mercaptoethanol and 500 µM spermidine. Spheroplasts were divided into 6 aliquots of 300 µL each and transferred into 1.5 mL Eppendorf tubes. Micrococcal nuclease (MNase; Worthington) dissolved in water at 0.1 U/µL stock was added to the tubes at concentrations of 0, 25, 50, 75, 100, and 150 U per sample. The digestion reactions were incubated at 37°C for 45 min and reactions were stopped by adding 75 µL of [5% SDS, 50 mM EDTA]. Remaining proteins were digested by adding 3 uL of 20 mg/mL proteinase K solution (Qiagen) to each tube for an overnight incubation at 65°C.

DNA from each MNase aliquot was isolated by phenol/chloroform extraction, concentrated via ethanol precipitation and treated with RNaseA (Fermentas) at a final concentration of 1 mg/ml for 1 hour. Samples were then separated on a 2% agarose gel and bands corresponding to ∼147 bp (mono-nucleosomal fragments) were gel-extracted using the QIAquick kit (Qiagen) for each of the digestions. Samples were then analyzed on the 2100 Agilent Bioanalyzer to determine the precise fragment size and quantity, and the samples with the greatest proportion of ∼147 bp fragments were used for hybridization.

### Total RNA isolation and cDNA synthesis

Isolation of total RNA and hybridization onto the tiling arrays followed (Juneau et al., 2007), except that Actinomycin D was added during cDNA synthesis to prevent antisense artifacts [75]. Briefly, RNA was isolated using the acid phenol method and treated with 1 U of DNase I mix (Invitrogen, Amp Grade) for 2 hours at 37°C. Cleanup was done using the RNeasy MinElute Cleanup Kit (Qiagen Cat. No. 74204). Single strand cDNA synthesis was performed using random primers in the presence of Actinomycin D in a final concentration of 6 µg/ml.

### Microarray labeling and hybridization

Nucleosomal DNA and cDNA samples were prepared in batches, where treatment conditions were paired with a wild-type sample that was harvested and prepared in parallel. Samples were fragmented by nuclease digestion in a solution containing 1× One-Phor-All buffer (GE Healthcare) and 1 µL of 1∶16 DNase I mix (Invitrogen, Amp Grade) at 37°C for 2 minutes, separated on a 2% agarose gel, and stained with SYBR green (Molecular Probes) to confirm that digestion produced a distribution of fragments less than 100 bp in size and a mean of 50 bp. These fragments were then labeled with terminal deoxynucleotidyl transferase (Amersham/GE Healthcare) and biotinylated ddATP (Perkin Elmer, NEL508) at 37°C for 2 hours, hybridized on Affymetrix arrays at 45°C for 24 hours, washed and stained according to protocol EukGE-WS2v4_450 in an Affymetrix Fluidics Station 450 and scanned in an Affymetrix 7G scanner (http://www.affymetrix.com/support/technical/fluidics_scripts.affx).

### Sequencing of mononucleosomal fragments

Preparation of Illumina libraries of mononucleosome fragments from the *rpb2-tet*, *rlf2-Δ*, *tup1-Δ*, *cyc8-Δ*, *spt16-ts*, *chd1-Δ*, *snf2-Δ*, *set2-Δ*, *swr1-Δ*, *msi1-Δ* and *tbf1-ts* strains was done according to [76]. Briefly, mononucleosome fragments were end-repaired, followed by amplification-free adaptor ligation and size selection on a 2% agarose gel. Clusters were generated on a single-read flowcell using Illumina's cBot, and sequenced as 2×36 nt paired-end reads using an Illumina Genome Analyzer IIx instrument. Read counts for each sample are provided in Table S5.

### Microarray and sequencing data processing

Mono-nucleosomal DNA tiling arrays from all mutant and wild-type control strains were quantile-normalized together with four independent monococcal nuclease-treated genomic DNA samples, using the AffyTiling package in Bioconductor [77]. A median smoothing filter was then applied to neighboring probes in the genome with a bandwidth of 30 nt, after which each array was rescaled to the same data range. Global variations in the baseline of the normalized signals were removed using waveNorm [78] with a window size of 5 kb. Genome-wide changes in nucleosome occupancy were assessed by comparing mutant strains to wild-type controls that were cultured and processed in parallel.

cDNA tiling arrays were quantile-normalized with Affymetrix Tiling Analysis Software (TAS) v1.1 using perfect-match probes only and a bandwidth of 30 nt. Raw data from cDNA hybridizations from mutant strains were normalized against cDNA from a wild-type control sample grown in parallel (mutant/WT). Data was visualized using the Integrated Genome Browser (IGB) [79].

For the nucleosome-sequencing experiments, paired-end sequencing reads were aligned to the *S. cerevisiae* genome (SGD, May 2008) using Bowtie v0.12.7 [80]. The midpoint of each mapped read pair was used as an estimate of a nucleosome center position.

### Nucleosome position analysis

A Gaussian filter (Matlab Statistics Toolbox) with a bandwidth of 50 nt, a mean of zero and a standard deviation of 25 was applied to the genomic-DNA normalized microarray intensity data, and nucleosome center positions were sequentially assigned to highest local maxima in the Gaussian score until no more peaks could be identified >100 nt away from already called positions [48]. The 10% nucleosome positions with the lowest Gaussian score were excluded from further analysis. For the nucleosome-sequencing experiments we determined nucleosome positions in the same way, except that the Gaussian filter was applied to counts of the number of mapped midpoints of paired-end nucleosomal reads at each position in the genome.

Nucleosome position shifts were assessed by comparing the wild-type midpoint position to that of the closest identified nucleosome position in the treatment condition within a 100 bp window around the WT condition. This window was conservatively defined to accommodate cases where an equivalent nucleosome could not be identified, e.g. because it was lost, which could otherwise lead to erroneous calculation of shifts relative to the positions of flanking nucleosomes. Our classification of nucleosome shifts is distinct from the concept of ‘positioning’ as defined by [81], which is a measure for how well-defined the position of a given nucleosome is in a population of cells. We identified a subset of significantly shifted nucleosomes that moved by at least 10 bp in the same direction in a replicate experiment, and had a P-value<0.05 in a T-test comparing the mean nucleosome position in each treatment to the mean across 31 wild-type conditions. genic nucleosomes were numbered relative to the curated transcription start site (TSS) of *S. cerevisiae* genes such that the +1 nucleosome is the first nucleosome with a center position downstream of the TSS. Positive and negative numbers correspond to shifts away or towards the TSS, respectively.

### Preparation of a curated set of *S. cerevisiae* transcript boundaries

A manually curated set of transcript starts and ends (Table S6) was prepared based on the May 2008 assembly from SGD, using a custom R script that allows for sequential assessment of *S. cerevisiae* transcripts in the context of whole-genome transcription and nucleosome occupancy data. Transcript boundaries were determined based on whole-genome tiling array hybridizations of Poly(A) and total RNA from wild-type strains in this study, which were quantile normalized using Affymetrix Tiling Array Software (v1.1), together with genomic DNA hybridizations as a control group to correct for cross-hybridization and probe GC content. Additional data sets included Poly(A) RNA-Seq data [82], [83], whole-genome Poly(A) tiling arrays [75] and whole-genome nucleosome occupancy data [11]. The nucleosome occupancy data was used to confirm the positions of the 5′ end of transcripts by assessing the presence of nucleosome-depleted regions. The R script and the data files used to determine transcript boundaries are provided as Dataset S1.

### Identification of genomic regions with transcription changes

Two-sided p-values were determined for each perfect-match probe using Affymetrix Tiling Analysis Software (TAS) v1.1 and a bandwidth of 30 bp, setting the mutant strains as the “treatment” and the WT strain as the “control” channel. Differentially transcribed regions were detected by setting combined thresholds for a p-value≤0.05 and a fold-change ≥2 in mutant compared to wild-type strains. These thresholds had to be met for a minimum of 80 nt (10 consecutive probes), with a maximum gap of 48 nt between probes exceeding this threshold. At these stringent settings some larger transcribed regions were detected as fragments and an additional step was therefore included to merge neighboring transcribed fragments (transfrags) if they met the following criteria: i) maximum gap of 250 bp; ii) mapped to the same strand; iii) same direction of change (both up or both down); iv) all intervening probes showed a consistent expression change in the same direction. To exclude known transcripts, transfrags that overlapped annotated coding and non-coding genes on the same strand for more than 20% of their length were removed from subsequent analyses. Remaining transfrags were classified as “tandem-sense”, “tandem-antisense”, “diverging”, “converging” or “intragenic antisense”, relative to overlapping and neighboring genes in a 3 kb region flanking the transfrag start.

### Histone level measurements

Each strain was grown in the exact same condition as described for the genome-wide assays of nucleosome occupancy and transcriptome profiling. 2.0 OD600 units of cells were pelleted, the supernatant removed and the cell pellets frozen. SDS-PAGE and immunoblotting was performed as described [84]. Extracts for immunoblotting were prepared from pelleted cells that were incubated in 0.5 mL of breaking solution (0.2 M NaOH, 0.2% β-mercaptoethanol) for 10 min on ice. Proteins were precipitated with 5% trichloracetic acid (final concentration), incubated for 10 min on ice, centrifuged in a microfuge at maximum speed for 5 min. Pellets were resuspended in 1× SDS loading buffer, incubated for 5 min at 95°C, and proteins separated on 10–20% SDS–polyacryamide gels. After electrotransfer, 0.22 micron nitrocellulose membranes (Invitrogen) were blocked for 1 h in TBST (10 mM Tris-HCl, 150 mM NaCl, 0.05% Tween 20 at pH 8.0) containing 5% dry milk powder. Antibodies used for immunoblotting were anti-histone H3 (Cell Signaling Technologies), anti-yeast PGK1 (Invitrogen), peroxidase anti-peroxidase (Sigma), developed with the Pierce Pico ECL substrate (Thermo). Blot bands were quantified using Image J [85] with the relative amount of histone protein (H3) compared to the appropriate wild-type or time 0 reference.

### qPCR for condition-specific changes in genes that are regulated by *ino80-Δ*

YJM789 wild-type cells and *ino80-Δ* mutant cells derived from this strain were cultured at 30°C in Yeast-Peptone-Dextrose (YPD) medium, YPGalactose medium or the presence of 1,10-phenanthroline. Cells were grown to mid-log phase (OD600∼0.8), collected by centrifuging at 3,000 rpm for 5 minutes and further grown in medium lacking the presence of glucose. Cells were collected by centrifuging at 3,000 rpm for 5 min and immediately frozen in liquid nitrogen, and stored at −80°C.

Total RNA was extracted from the cells. Reverse transcription was performed with the SuperScript™ II Reverse Transcriptase from Invitrogen. In brief, 25 ng of total RNA was used as the starting material for single strand synthesis in the presence of Actinomycin D. Quantitative PCR was performed with the SYBR Green PCR Master Mix (Applied Biosystems) in an Applied Biosystems 7900HT Fast Real-Time PCR System using Sequence Detection System software version 2.3. The following temperature profile was applied: 50°C for 2 min; 95°C for 15 min; 40 cycles of the sequence 95°C for 15 s; 58°C for 1 min; 95°C for 15 s; 60°C for 15 s; 95°C for 15 s. Primer pairs used were 5′-CCTGAACAGAAACAACTACAA-3′ and 5′-CCATTGTTGTTTGGATTTGCATTG-3′ for *MIG1*, 5′ GCCAGGTGGCAAGTTTTTCG-3′, 5′-CTGTACTTCATTATGTGAATCACG-3′ for *RGT1*, 5′ GTA TAG TGG CAA CGA TAT TAA TGT C 3′ and 5′ GTA CTA CCA TTG TCA CAC CC 3′ for *FRE1*, and 5′-CCTGGTAGAAGTTTCATGGC-3′ and 5′-GGTCAACACATATTCGTGAGAAC-3′ for *FRE7*. Fold change in the *MIG1*, *RGT1*, *FRE1* and *FRE7* transcript level normalized to *ACT1* (5′-TGTGATGTCGATGTCCGTAAG-3′ and 5′-CGGTGATTTCCTTTTGCATT-3′) was calculated using the 2−ΔΔCt method. At least three independent replicates of each reaction were performed. Student's t-test was applied for statistical analysis (paired for control vs. treatments, and unpaired for mutants vs. wild type).

### *TCR* deletions and spot assays under stress conditions

Deletion of a selection of *TCR* genes, *TCR01*, *TCR03*, *TCR05*, *TCR06*, *TCR08*, *TCR11*, *TCR15*, *TCR20*, *TCR21*, *TCR22*, *TCR27*, *TCR29*, *TCR30* and *TCR31* in the strains *ura8-*Δ and *tup1-*Δ were created using a PCR-based gene deletion strategy to generate a start- to stop- codon deletion of each of the genes in the yeast genome (Table S7). As part of the deletion process, each gene disruption was replaced with a *NATR* module. Deletions were verified using confirmation primers (Table S7). *TCR* deletions in the *ura8-*Δ and *tup1-*Δ background were then grown in a wide variety of stress conditions and a spot assay was done in the presence of 5 mM EDTA and NaCl to avoid the tup1 flocculation phenotype. Spot assays were performed on 30°C YPD, 37°C YPD, 0.1 mM H_2_O_2_, 1 M NaCl, 5% ethanol, 3% ethanol, 1% ethanol, YPGlyercol, YPGalactose, 5 mM DTT, 15 mM DTT, 0.7 mM paraquat, 1 mg/ml 6-azauracil and 5 mg/ml 6-azauracil. To determine the growth defect for the spot dilution assays on various stress conditions, high-resolution images were taken of each strain and by using ImageJ (v1.45) to determine the intensity profile of the pixels that present the yeast colonies.

### Accession numbers

Affymetrix tiling array data and next-generation sequencing data are available at GEO (record GSE44879).

## Supporting Information

Dataset S1 Yeast transcript annotation R script and data files. Please note this file is 81.9 MB and may be difficult for some readers to download due to its size.
(GZ) Click here for additional data file.

Figure S1 Nucleosome occupancy profiles for all compendium conditions. Intensity plots of nucleosome occupancy relative to the curated transcription start sites of 5,043 *S. cerevisiae* genes, expressed as the log_2_ ratio of probe intensities of MNase-treated nucleosomal DNA samples over MNase-treated genomic DNA.
(PDF)

Figure S2 Correlation between NDR occupancy changes and gene expression changes in transcription factor mutants. Hierarchical clustering of NDR nucleosome occupancy changes ≥1.5 fold in at least one of the transcription factor loss-of-function mutants included in the compendium (left). Expression changes for the genes associated with each NDR are shown in comparison (right). NDRs were defined as the 200 bp region directly upstream of curated transcription start sites.
(PDF)

Figure S3 Selection of nucleosome shift profiles. Nucleosome shift profiles for representative essential (A) and non-essential (B) loss-of-function mutants, drug treatments (C) and wild-type (WT) reference strains (D). The position changes in the 5 WT strains are plotted relative to the median nucleosome position across all 35 WT reference profiles used in this study.
(PDF)

Figure S4 Remodeler ATPase binding is not increased at positions with nucleosome shifts in deletion mutants. WT (top row) and Ino80 and Isw1 deletion mutant (middle row) occupancy profiles at genic nucleosome positions are shown in comparison to MNase-ChIP binding profiles for these factors at the same locations (bottom row). MNase-ChIP data were obtained from Yen et al. [36]. Plots were prepared are as described in Figure 4A.
(PDF)

Figure S5 Changes in global histone H3 levels for selected compendium mutants. Change in histone H3 levels in 11 compendium conditions compared to wild-type controls. Each strain was grown in the exact same condition as described for the genome-wide assays of nucleosome occupancy and transcriptome profiling. Equal amount of OD units were loaded for each mutant and histone H3 bands were quantified from western blots using Image J. Error bars correspond to the standard deviation of the changes in normalized histone levels in three western blot replicates, using PGK1 as a loading control for normalization.
(PDF)

Figure S6 Nucleosomes disrupted after 6 hours of histone depletion and after loss of Spt16 function assume more intrinsically preferred positions. A) Occupancy profiles at genic nucleosome positions in WT conditions (top row) and after 3–6 hours of histone depletion or loss of Spt16 (middle row) are shown in comparison to predicted occupancy profiles (Lasso model score) based on intrinsic sequence preferences obtained from Kaplan et al. [27] (bottom row). B) Effects of nucleosome shifts on neighboring nucleosomes. Plots were prepared are as described in Figure 3.
(PDF)

Figure S7 Reanalysis of previously published histone depletion and reconstitution data reveals shifts of proximal genic nucleosomes. Nucleosome shifts in nhp6 mutants compared to wild-type strains [59] (green) and between *in vitro* reconstituted nucleosomes with 0.5∶1 and 1∶1 histone∶DNA ratios (red) [33]. Profiles were prepared as described in Figure 2.
(PDF)

Figure S8 Nucleosomes disrupted in CAF-1 complex mutants assume more intrinsically preferred positions. A) Cac2, Msi1 and Rlf2 WT (top row) and deletion mutant (middle row) occupancy profiles at genic nucleosome positions are shown in comparison to predicted occupancy profiles (Lasso model score) based on intrinsic sequence preferences obtained from Kaplan et al. [27] (bottom row). B) Effects of nucleosome shifts on neighboring nucleosomes. Plots were prepared are as described in Figure 3.
(PDF)

Figure S9 Large changes in NDR nucleosome occupancy 6 hours after histone H4 transcription shutoff do not result in cryptic promoter transcripts. Correlation between changes in promoter nucleosome occupancy (left panels, yellow/blue) and transcription changes across the gene body and 1 kb intergenic flanking regions (right panels, red/green). In each panel, genes are ranked according to the average change in NDR nucleosome occupancy.
(PDF)

Table S1 Strain information.
(XLS)

Table S2 Listing of antisense and intergenic regions with significant expression changes.
(XLS)

Table S3 PATs identified in Abf1, Rap1, Rsc3 and Tbf1 loss-of-function mutants.
(XLS)

Table S4 Genomic locations of Tup1/Cyc8 repressed (TCR) transcripts.
(XLS)

Table S5 Nucleosome-Seq read counts.
(XLS)

Table S6 List of manually curated gene starts and ends.
(XLS)

Table S7 Deletion and confirmation primers for *TCR* gene deletions.
(XLS)
