# Supplementary material for: A Compendium of Nucleosome and Transcript Profiles Reveals Determinants of Chromatin Architecture and Transcription
Source: PLoS Genet. 2013 May 2;9(5):e1003479. doi: 10.1371/journal.pgen.1003479 (PMC3642058; doi:10.1371/journal.pgen.1003479)
Supplement: Dataset S1 — Yeast transcript annotation R script and data files. Please note this file is 81.9 MB and may be difficult for some readers to download due to its size. (GZ) [file pgen.1003479.s001.gz › yeast-transcript-annotation/README.pdf]

Instructions for annotating yeast transcripts

The archive contains a set of datafiles for each yeast chromosomes as well as the analysis script itself (annotate-transcripts.R) and this readme. To do the analysis you first need to install R, which can be downloaded for free from http://www.r-project.org/. After starting R you have to set the working directory for R, which has to point to the directory with the R script and data files,by entering the following in the R command window:

```
setwd(c:/path/to/directory)
```

Note that paths in R need to be specified with forward slashes. Alternatively, on mac or windows you can select 'set current working directory' from one of the menu options. Once the working directory has been set up, load the analysis functions by entering:

```
source('annotate-transcripts.R')
```

Starting the annotation

The first step in annotating the transcripts is loading the files with the intensity data, sequencing calls and ORF annotations. Enter the following command, replacing **N** by the chromosome you want to analyze.

```
data.chrN = read.files('chrN')
```

You can now start annotating by entering the following command, again replacing 'N' by the number of the chromosome you just loaded the data for:

```
annotations.chrN = annotate.transcripts(data.chrN)
```

A plot window will open, showing the first ORF on the chromosome (example below). The new annotations will be saved to the object '`annotations.chrN`'.

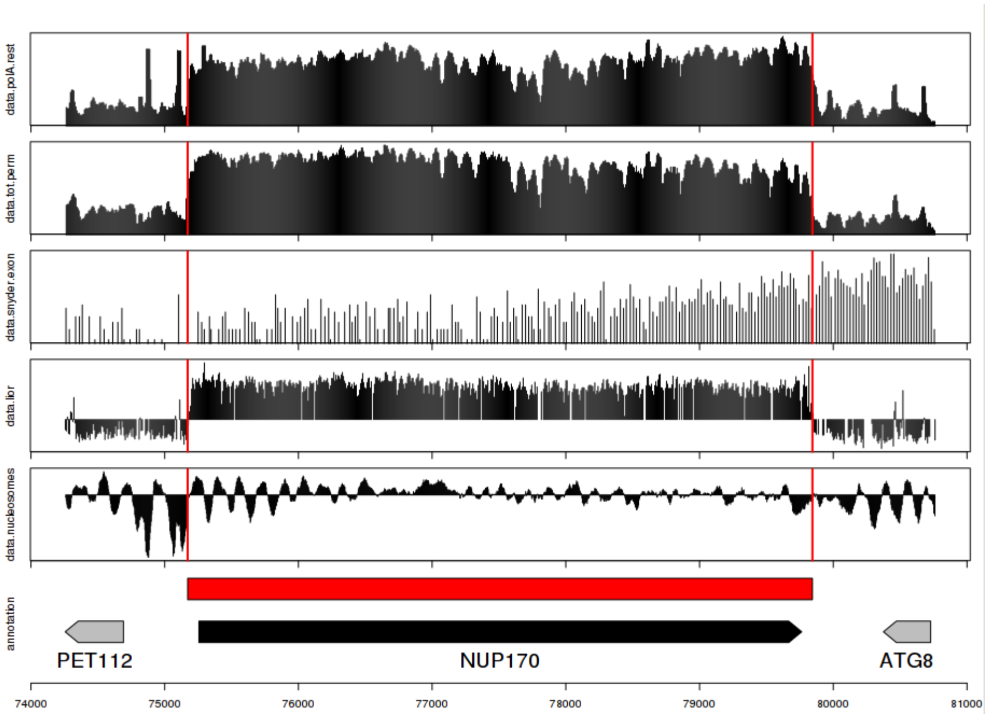

The first two tracks in the window are PolA+ and Total RNA hybridizations, respectively. For each graph, the vertical bars indicates the expression signal. The third track is Mike Snyder's sequencing data (http://www.sciencemag.org/cgi/content/full/320/5881/1344). The bars represent counts binned at 20 bp intervals. The fourth track is data from Lior et al. (http://nar.oxfordjournals.org/cgi/content/full/gkm683v1). The final track is the nucleosome occupancy data from Lee et al.

Some characteristics for each dataset:

- PolyA+ hyb, Toronto
  - PolyA-primed so better definition of 3' ends
- Total RNA Hyb, Toronto
  - Random primed only. Cleaner hybs but less well defined 3' ends
- Snyder lab sequencing data
  - Vertical bars indicate the log2 of the number of counts in a 20-bp window
  - A lot of ORFs are poorly defined by sequence reads (insufficient coverage), but the sequencing data often helps to define 3' ends
  - The blue dots correspond to reads that had a A/T composition that suggests a polyadenylation site. This data is not completely accurate, but in cases where there are multiple 3' end counts (cluster of dots or dots with high count) it helps to define the actual stop site.
- Lior data
  - The lior data is GC-corrected and problematic probes were removed. This helps delineate the transcript start/ends most cases, but not all.

The annotation itself is mostly mouse-click driven. Two left-clicks will set the transcription start and end (the mouse click order, start-end or end-start, is not important), after the second click, the window will refresh and a red box and vertical red lines in each graph will display the selected start and end site. If you are not satisfied with the positioning, you can simply give another two left-clicks to set a new start and end. When you are satisfied with the annotation, right-click once to store the annotation data and proceed to the next ORF.

Finally, if you use the combination left-click followed by a right-click, you will get an options menu in the R command window. This will allow you to delete the current annotation or to stop annotating halfway the chromosome (and continue later).

Resuming or reviewing annotations

To resume or review an annotation that was halted in the middle, simply issue the following command:

```
annotations.chrN = annotate.transcripts(data.chrN, annotations.chrN)
```

This will display all ORFs again together with transcript info for the ones you annotated before. You can skip through the ones that are already annotated by right-clicking through them.

Saving the annotation data
To save the annotation data as an R object, issue the following command (obviously replacing **N** again with the chromosome number):

```
save(annotations.chrN, file='annotations.chrN.Rdata')
```

This will save a file called "annotations.chr**N**.Rdata' in the same directory where you unzipped the original files.

Additionally, you can save the data as a gff-formatted file that you can load into IGB or use to generate feature matrices & average plots. The command is:

```
write.mappings.file(annotations.chrN, file='annotations.chrN.gff')
```
