# Supplementary figures and images for: A Compendium of Nucleosome and Transcript Profiles Reveals Determinants of Chromatin Architecture and Transcription
Source: PLoS Genet. 2013 May 2;9(5):e1003479. doi: 10.1371/journal.pgen.1003479 (PMC3642058; doi:10.1371/journal.pgen.1003479)

Figure S1

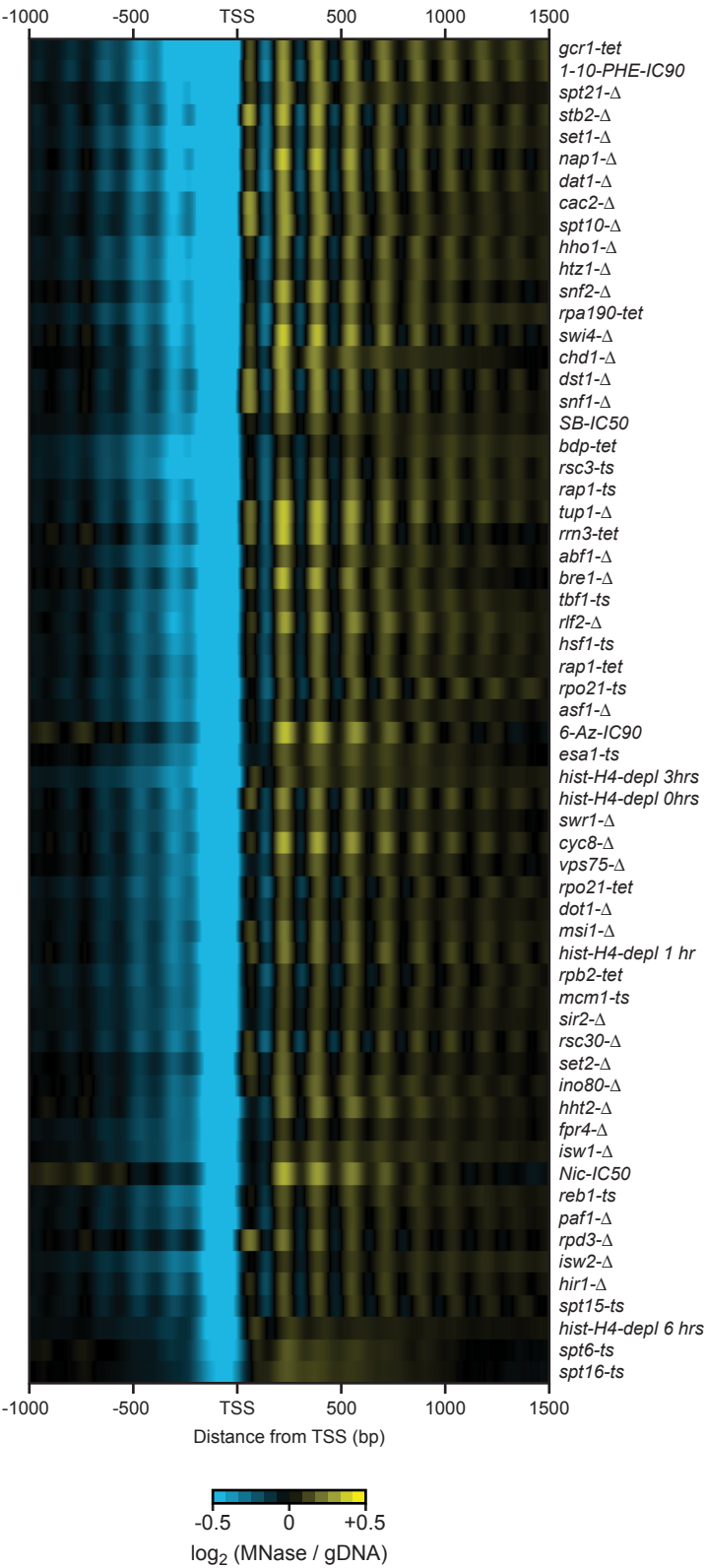

Supplement: Figure S1 — Nucleosome occupancy profiles for all compendium conditions. Intensity plots of nucleosome occupancy relative to the curated transcription start sites of 5,043 S. cerevisiae genes, expressed as the log2 ratio of probe intensities of MNase-treated nucleosomal DNA samples over MNase-treated genomic DNA. (PDF) [file pgen.1003479.s002.pdf]

Figure S2

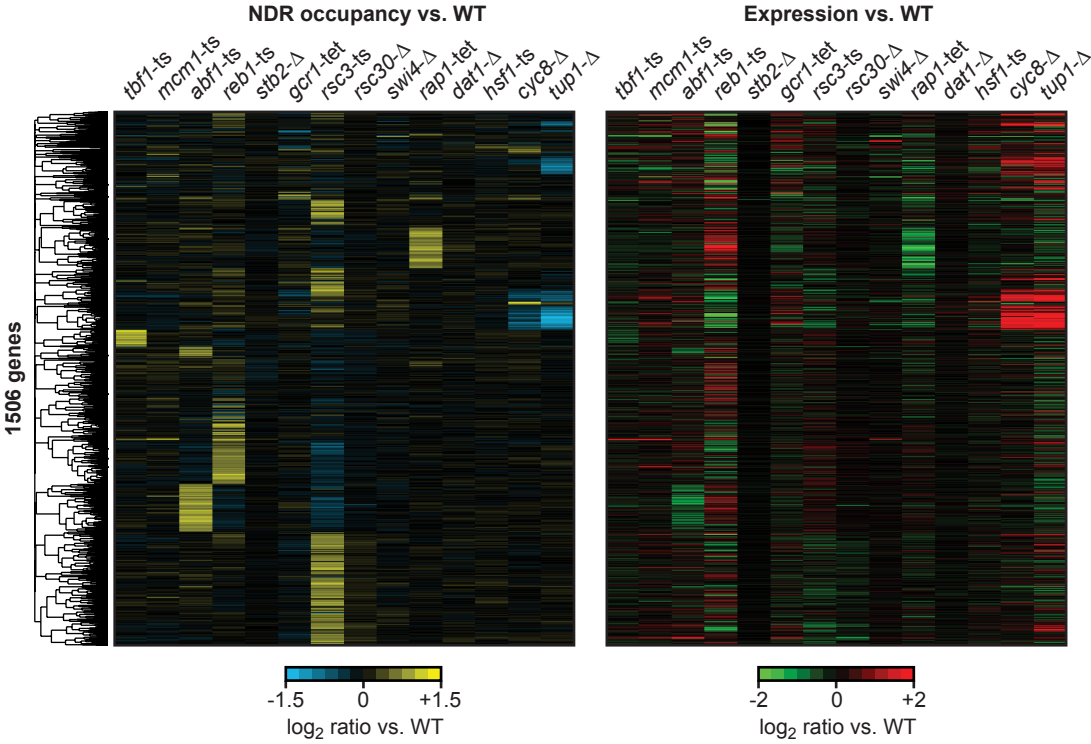

Supplement: Figure S2 — Correlation between NDR occupancy changes and gene expression changes in transcription factor mutants. Hierarchical clustering of NDR nucleosome occupancy changes ≥1.5 fold in at least one of the transcription factor loss-of-function mutants included in the compendium (left). Expression changes for the genes associated with each NDR are shown in comparison (right). NDRs were defined as the 200 bp region directly upstream of curated transcription start sites. (PDF) [file pgen.1003479.s003.pdf]

**Figure S3**

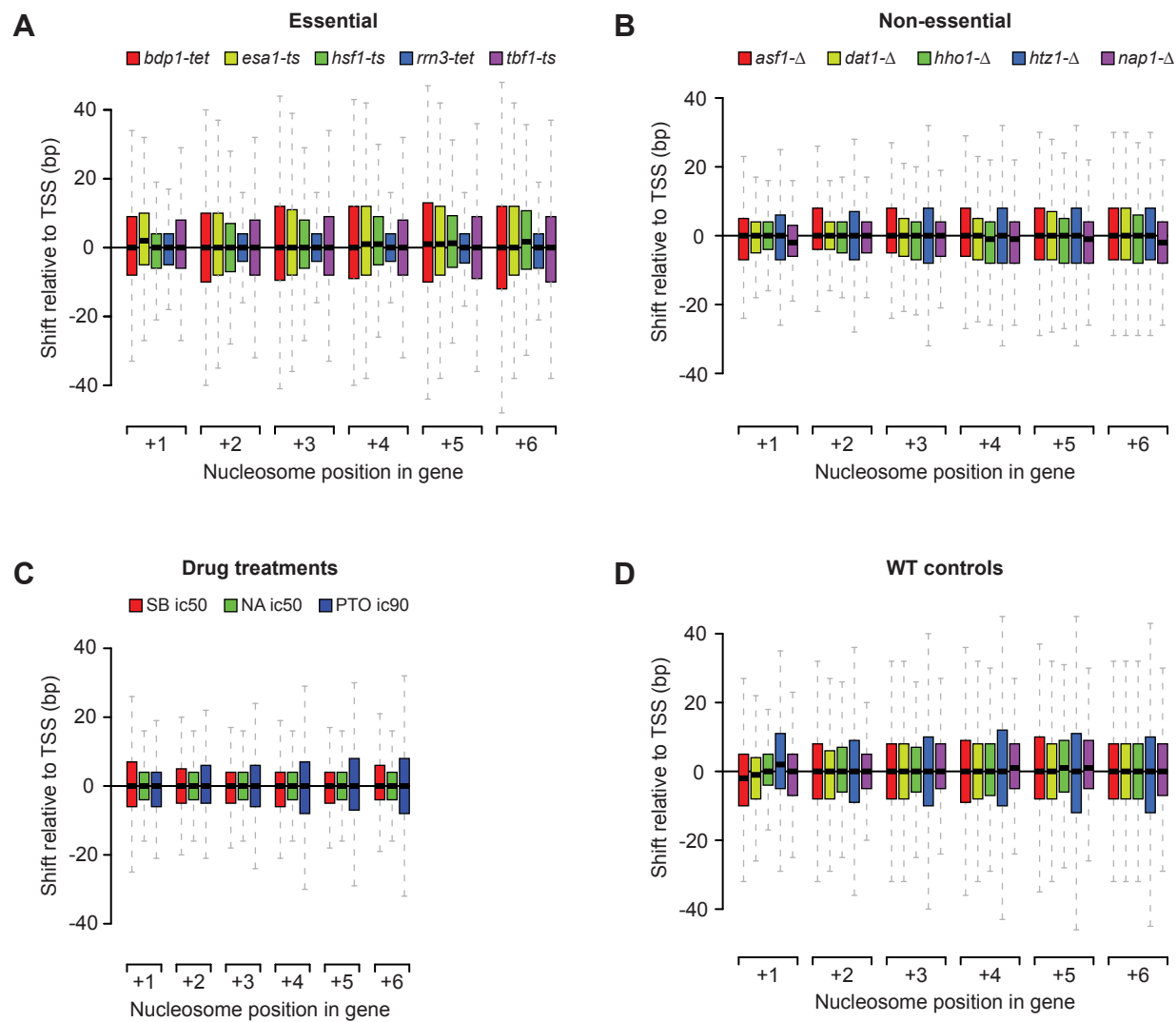

Supplement: Figure S3 — Selection of nucleosome shift profiles. Nucleosome shift profiles for representative essential (A) and non-essential (B) loss-of-function mutants, drug treatments (C) and wild-type (WT) reference strains (D). The position changes in the 5 WT strains are plotted relative to the median nucleosome position across all 35 WT reference profiles used in this study. (PDF) [file pgen.1003479.s004.pdf]

Figure S4

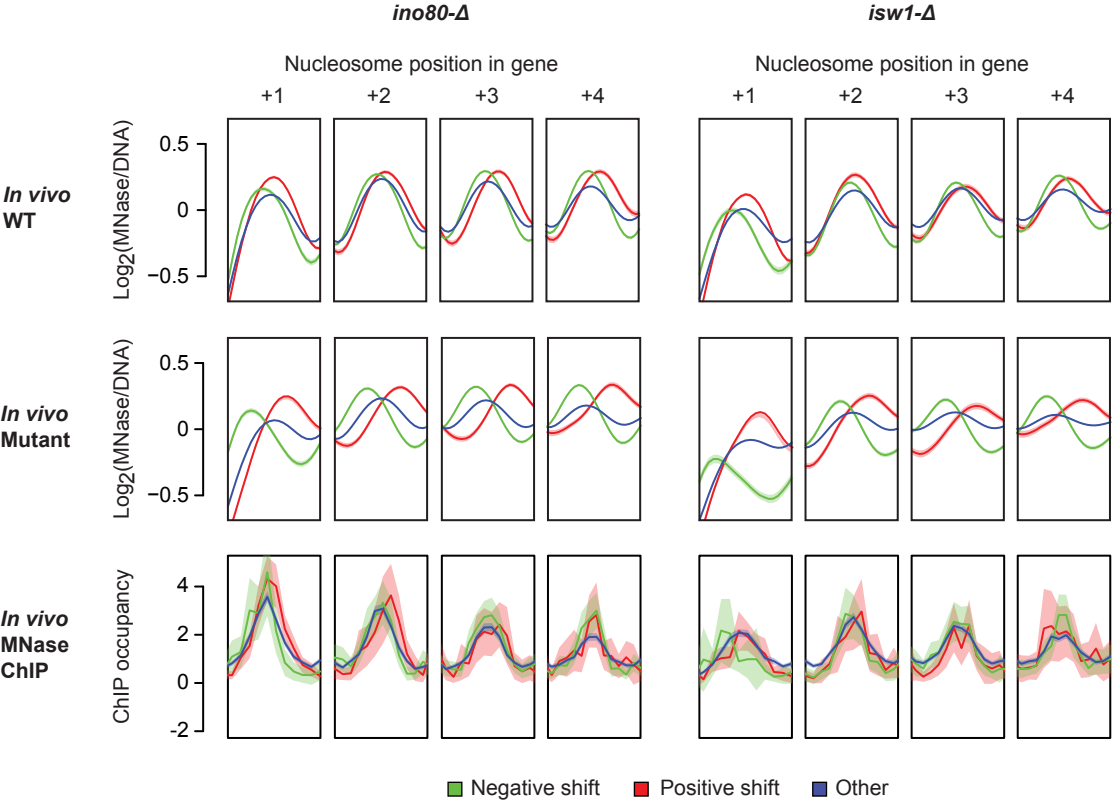

Supplement: Figure S4 — Remodeler ATPase binding is not increased at positions with nucleosome shifts in deletion mutants. WT (top row) and Ino80 and Isw1 deletion mutant (middle row) occupancy profiles at genic nucleosome positions are shown in comparison to MNase-ChIP binding profiles for these factors at the same locations (bottom row). MNase-ChIP data were obtained from Yen et al. [36]. Plots were prepared are as described in Figure 4A. (PDF) [file pgen.1003479.s005.pdf]

**Figure S5**

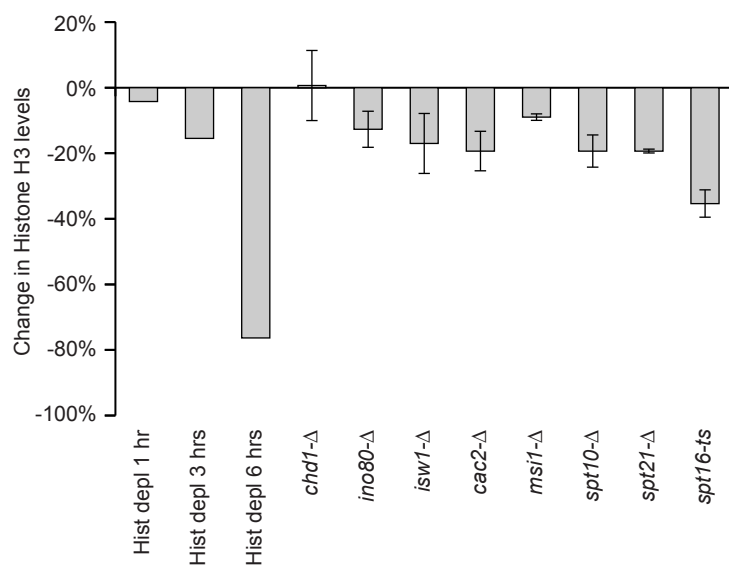

Supplement: Figure S5 — Changes in global histone H3 levels for selected compendium mutants. Change in histone H3 levels in 11 compendium conditions compared to wild-type controls. Each strain was grown in the exact same condition as described for the genome-wide assays of nucleosome occupancy and transcriptome profiling. Equal amount of OD units were loaded for each mutant and histone H3 bands were quantified from western blots using Image J. Error bars correspond to the standard deviation of the changes in normalized histone levels in three western blot replicates, using PGK1 as a loading control for normalization. (PDF) [file pgen.1003479.s006.pdf]

Figure S6

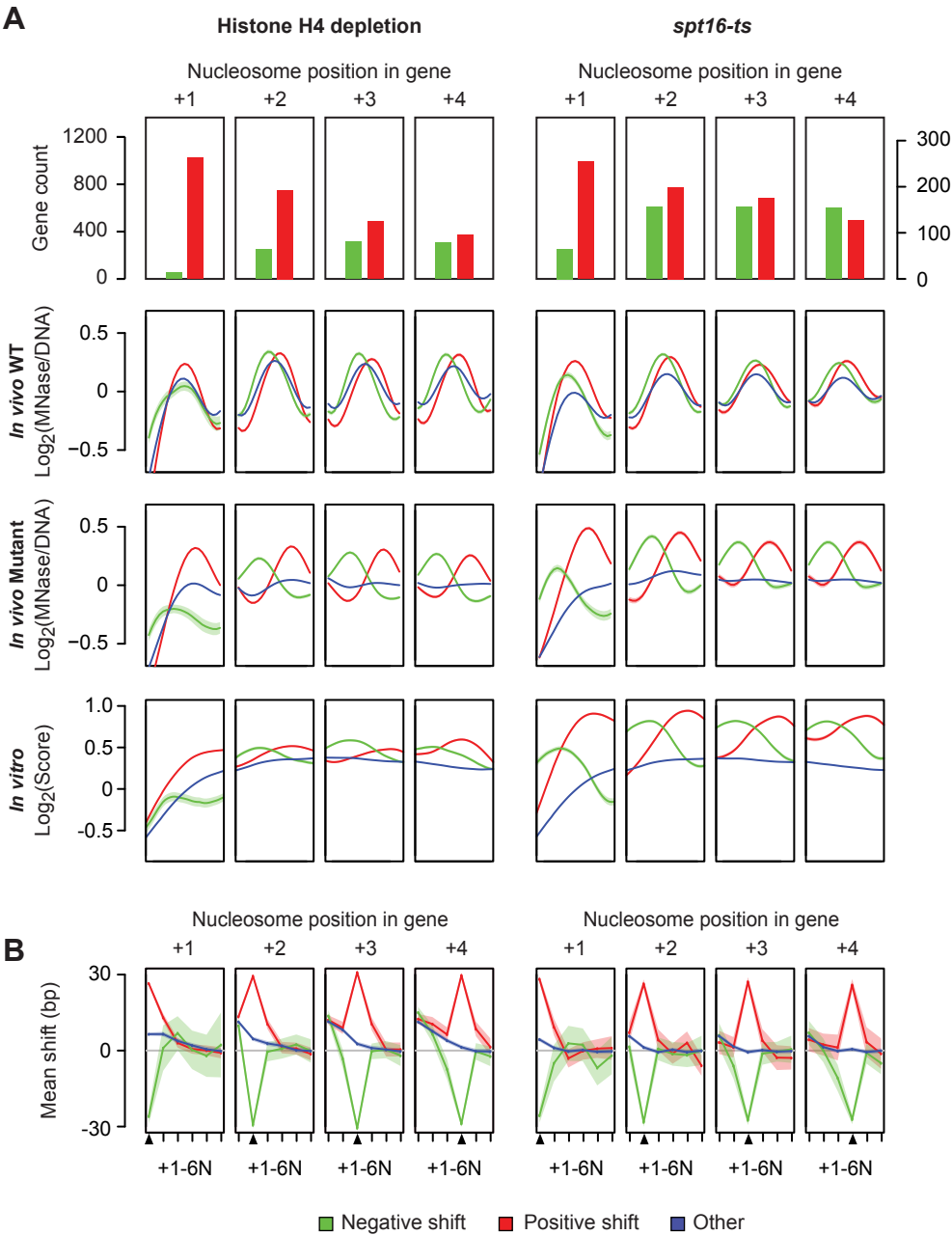

Supplement: Figure S6 — Nucleosomes disrupted after 6 hours of histone depletion and after loss of Spt16 function assume more intrinsically preferred positions. A) Occupancy profiles at genic nucleosome positions in WT conditions (top row) and after 3–6 hours of histone depletion or loss of Spt16 (middle row) are shown in comparison to predicted occupancy profiles (Lasso model score) based on intrinsic sequence preferences obtained from Kaplan et al. [27] (bottom row). B) Effects of nucleosome shifts on neighboring nucleosomes. Plots were prepared are as described in Figure 3. (PDF) [file pgen.1003479.s007.pdf]

Figure S7

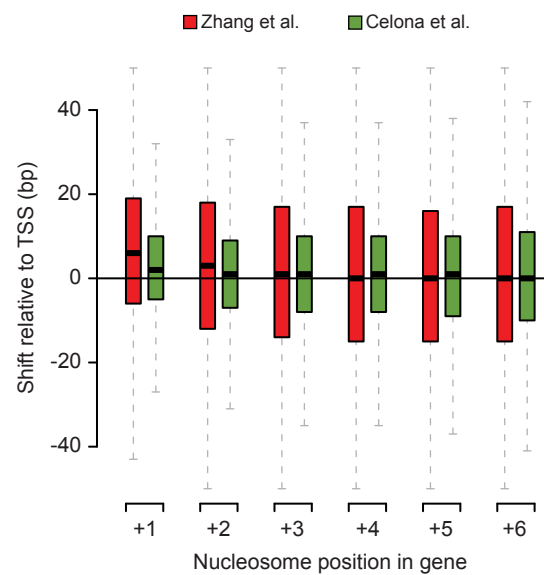

Supplement: Figure S7 — Reanalysis of previously published histone depletion and reconstitution data reveals shifts of proximal genic nucleosomes. Nucleosome shifts in nhp6 mutants compared to wild-type strains [59] (green) and between in vitro reconstituted nucleosomes with 0.5∶1 and 1∶1 histone∶DNA ratios (red) [33]. Profiles were prepared as described in Figure 2. (PDF) [file pgen.1003479.s008.pdf]

Figure S8

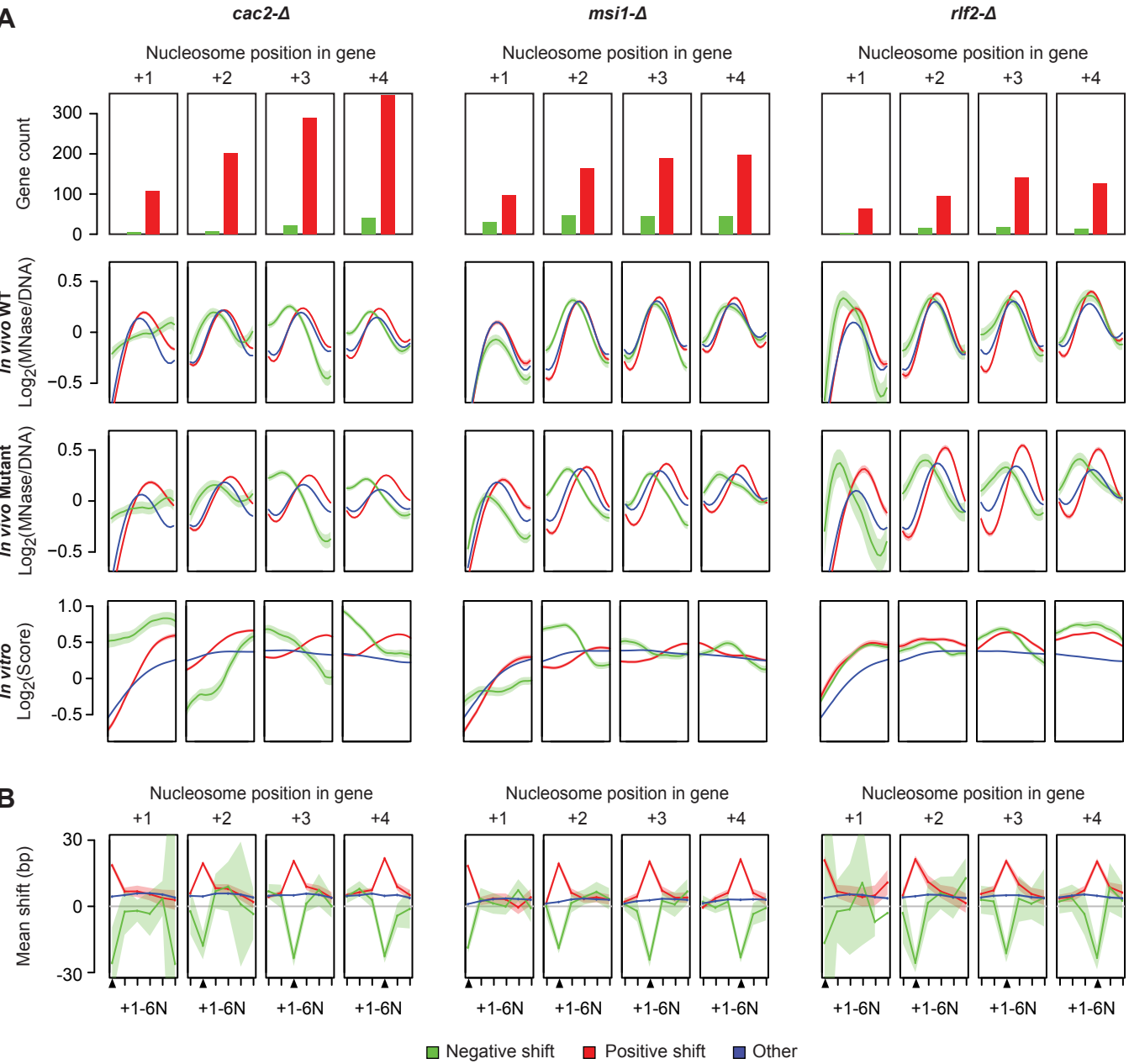

Supplement: Figure S8 — Nucleosomes disrupted in CAF-1 complex mutants assume more intrinsically preferred positions. A) Cac2, Msi1 and Rlf2 WT (top row) and deletion mutant (middle row) occupancy profiles at genic nucleosome positions are shown in comparison to predicted occupancy profiles (Lasso model score) based on intrinsic sequence preferences obtained from Kaplan et al. [27] (bottom row). B) Effects of nucleosome shifts on neighboring nucleosomes. Plots were prepared are as described in Figure 3. (PDF) [file pgen.1003479.s009.pdf]

**Figure S9**

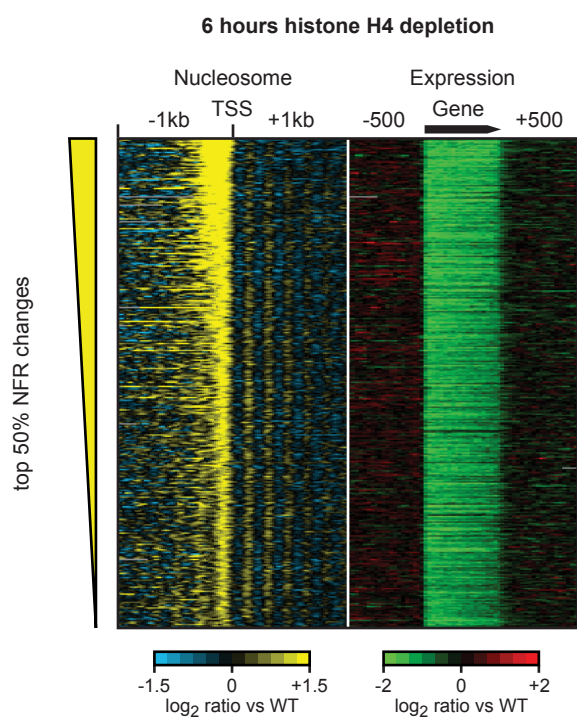

Supplement: Figure S9 — Large changes in NDR nucleosome occupancy 6 hours after histone H4 transcription shutoff do not result in cryptic promoter transcripts. Correlation between changes in promoter nucleosome occupancy (left panels, yellow/blue) and transcription changes across the gene body and 1 kb intergenic flanking regions (right panels, red/green). In each panel, genes are ranked according to the average change in NDR nucleosome occupancy. (PDF) [file pgen.1003479.s010.pdf]
